# PARP4 interacts with hnRNPM to regulate splicing during lung cancer progression

**DOI:** 10.1186/s13073-024-01328-1

**Published:** 2024-07-22

**Authors:** Yi Fei Lee, Cheryl Zi Jin Phua, Ju Yuan, Bin Zhang, May Yin Lee, Srinivasaraghavan Kannan, Yui Hei Jasper Chiu, Casslynn Wei Qian Koh, Choon Kong Yap, Edwin Kok Hao Lim, Jianbin Chen, Yuhua Lim, Jane Jia Hui Lee, Anders Jacobsen Skanderup, Zhenxun Wang, Weiwei Zhai, Nguan Soon Tan, Chandra S. Verma, Yvonne Tay, Daniel Shao Weng Tan, Wai Leong Tam

**Affiliations:** 1 Genome Institute of Singapore (GIS), Agency for Science, Technology and Research (A*STAR), 60 Biopolis Street, Genome, Singapore, 138672 Singapore; 2 School of Biological Sciences, Nanyang Technological University, 60 Nanyang Drive, Singapore, 637551 Singapore; 3 Cancer Science Institute of Singapore, National University of Singapore, 14 Medical Drive, Singapore, 117599 Singapore; 4 Computational Bioscience Research Center, King Abdullah University of Science and Technology (KAUST), Thuwal, Saudi Arabia; 5 Computer Science Program, Computer, Electrical and Mathematical Sciences and Engineering Division, King Abdullah University of Science and Technology (KAUST), Thuwal, Saudi Arabia; 6 Bioinformatics Institute (BII), Agency for Science, Technology and Research (A*STAR), 30 Biopolis Street, Matrix, Singapore, 138671 Singapore; 7 Centre for Vision Research, Duke-NUS Medical School, 8 College Road, Singapore, 169857 Singapore; 8 Key Laboratory of Zoological Systematics and Evolution, Institute of Zoology, Chinese Academy of Sciences, Beijing, China; 9 Center for Excellence in Animal Evolution and Genetics, Chinese Academy of Sciences, Kunming, China; 10 Lee Kong Chian School of Medicine, Nanyang Technological University, 11 Mandalay Road, Singapore, 308232 Singapore; 11 Department of Biological Sciences, National University of Singapore, 16 Science Drive 4, Singapore, 117558 Singapore; 12 NUS Centre for Cancer Research, Yong Loo Lin School of Medicine, National University of Singapore, 14 Medical Drive, Singapore, 117599 Singapore; 13 Department of Biochemistry, Yong Loo Lin School of Medicine, National University of Singapore, 8 Medical Drive, Singapore, 117597 Singapore; 14 Division of Medical Oncology, National Cancer Centre Singapore, 30 Hospital Boulevard, Singapore, 168583 Singapore

**Keywords:** Non-small-cell lung cancer, Functional genomics, Mechanisms of disease

## Abstract

**Background:**

The identification of cancer driver genes from sequencing data has been crucial in deepening our understanding of tumor biology and expanding targeted therapy options. However, apart from the most commonly altered genes, the mechanisms underlying the contribution of other mutations to cancer acquisition remain understudied. Leveraging on our whole-exome sequencing of the largest Asian lung adenocarcinoma (LUAD) cohort (*n* = 302), we now functionally assess the mechanistic role of a novel driver, PARP4.

**Methods:**

In vitro and in vivo tumorigenicity assays were used to study the functional effects of PARP4 loss and mutation in multiple lung cancer cell lines. Interactomics analysis by quantitative mass spectrometry was conducted to identify PARP4’s interaction partners. Transcriptomic data from cell lines and patient tumors were used to investigate splicing alterations.

**Results:**

PARP4 depletion or mutation (I1039T) promotes the tumorigenicity of KRAS- or EGFR-driven lung cancer cells. Disruption of the vault complex, with which PARP4 is commonly associated, did not alter tumorigenicity, indicating that PARP4’s tumor suppressive activity is mediated independently. The splicing regulator hnRNPM is a potentially novel PARP4 interaction partner, the loss of which likewise promotes tumor formation. hnRNPM loss results in splicing perturbations, with a propensity for dysregulated intronic splicing that was similarly observed in PARP4 knockdown cells and in LUAD cohort patients with PARP4 copy number loss.

**Conclusions:**

PARP4 is a novel modulator of lung adenocarcinoma, where its tumor suppressive activity is mediated not through the vault complex—unlike conventionally thought, but in association with its novel interaction partner hnRNPM, thus suggesting a role for splicing dysregulation in LUAD tumorigenesis.

**Supplementary Information:**

The online version contains supplementary material available at 10.1186/s13073-024-01328-1.

## Background

Lung cancer is among the most frequently occurring cancers and a leading cause of cancer mortality worldwide [1]. Among lung cancer cases, lung adenocarcinoma (LUAD) presents as the most prevalent histologic subtype, accounting for 38.5% of all cases [2]. Genomic studies dissecting the mutational landscape of LUAD have been pivotal in shedding light on the characteristics of LUAD, allowing for the identification of biomarkers and treatment options [3]. The discovery of activating EGFR mutations has heralded an era of precision oncology, with several generations of EGFR kinase inhibitors such as gefitinib and osimertinib now available [4]. Much, however, remains to be explored in terms of the functional relevance of other mutations uncovered from these studies, which may in turn provide a better understanding of LUAD pathogenesis and offer new therapeutic opportunities.

LUAD genomic studies have also precipitated the recognition of ethnic variation in driver gene mutational frequencies. Notably, EGFR mutations are present in 40–60% of Asian LUAD cases but make up only 7–10% of Caucasian LUAD cases, among which KRAS mutations are more prevalent [5]. Apart from differences in molecular profiles, Caucasian LUAD tends to be dominated by male smokers, whereas LUAD in East Asians is enriched with female non-smokers [6]. LUAD in East Asians also tends to have an early onset, especially in non-smokers [7]. Other epidemiological differences between Caucasian and Asian LUAD take the form of differing incidence rates, risk factors, responses to targeted therapies, and prognoses [8]. Recently, we performed whole-exome and RNA sequencing on the largest Asian LUAD cohort to date [9–11], with the aim of better characterizing the genomic and transcriptomic landscape of Asian LUAD. Specifically, this Asian LUAD cohort comprised 210 ethnically Chinese Singaporean LUAD patients and included 92 Chinese patients from an independently published study [12]. This dataset, which was inclusive of male and female smokers and non-smokers across all stages, provided a useful resource in identifying the distinctive genetic features between Asian and Caucasian LUAD.

Given that understanding the mutational landscape of LUAD has enabled the identification of important therapeutic targets such as EGFR and ALK, we sought to examine this within the Asian cohort. Two computational driver prediction approaches—MutSigCV and 20/20 + —were applied to the whole-exome sequencing data from the LUAD cohort and identified several potential new modulators of LUAD that are mutated in patients, in which their precise functions are unknown [9, 10]. In this study, we focused on examining the role of PARP4, which was found to be important in protecting against LUAD disease progression. PARP4 is a member of the family of poly-ADP-ribose polymerases that catalyze reversible ADP-ribosylation [13]. PARP4 was first discovered as a component of the vault complex, which is a barrel-shaped ribonucleoprotein assembly comprising an outer shell formed by major vault protein (MVP) monomers enclosing PARP4, telomerase-associated protein-1 (TEP1), and short vault RNAs within [14]. Specifically, the vault complex has been proposed to mediate nucleocytoplasmic transport of cargo [15], drug resistance [16], and signal transduction by serving as a protein scaffold [17], although the contribution of PARP4 to these phenotypes remains to be determined. Notably, fractionation and immunofluorescence experiments revealed that PARP4’s subcellular distribution overlaps only partially with that of MVP, with non-vault-associated fractions of PARP4 having been reported in both the cytoplasm and nucleus [14, 18]. The functional significance of these PARP4 fractions remains to be understood.

PARP4 function appears to be dispensable under normal contexts as genetically engineered mice with PARP4 deficiency are viable and fertile. When challenged with a chemical carcinogen, however, these PARP4-deficient mice were more prone to developing dimethylhydrazine-induced colon tumors [19, 20]. A few studies also reported germline PARP4 mutations in cancer patients, and PARP4 was proposed as a candidate cancer susceptibility gene [21–24]. Here we elucidate the functional effects of PARP4 on LUAD and highlight the potential contribution of splicing.

## Methods

### Cohort data

**Fig. 1:**
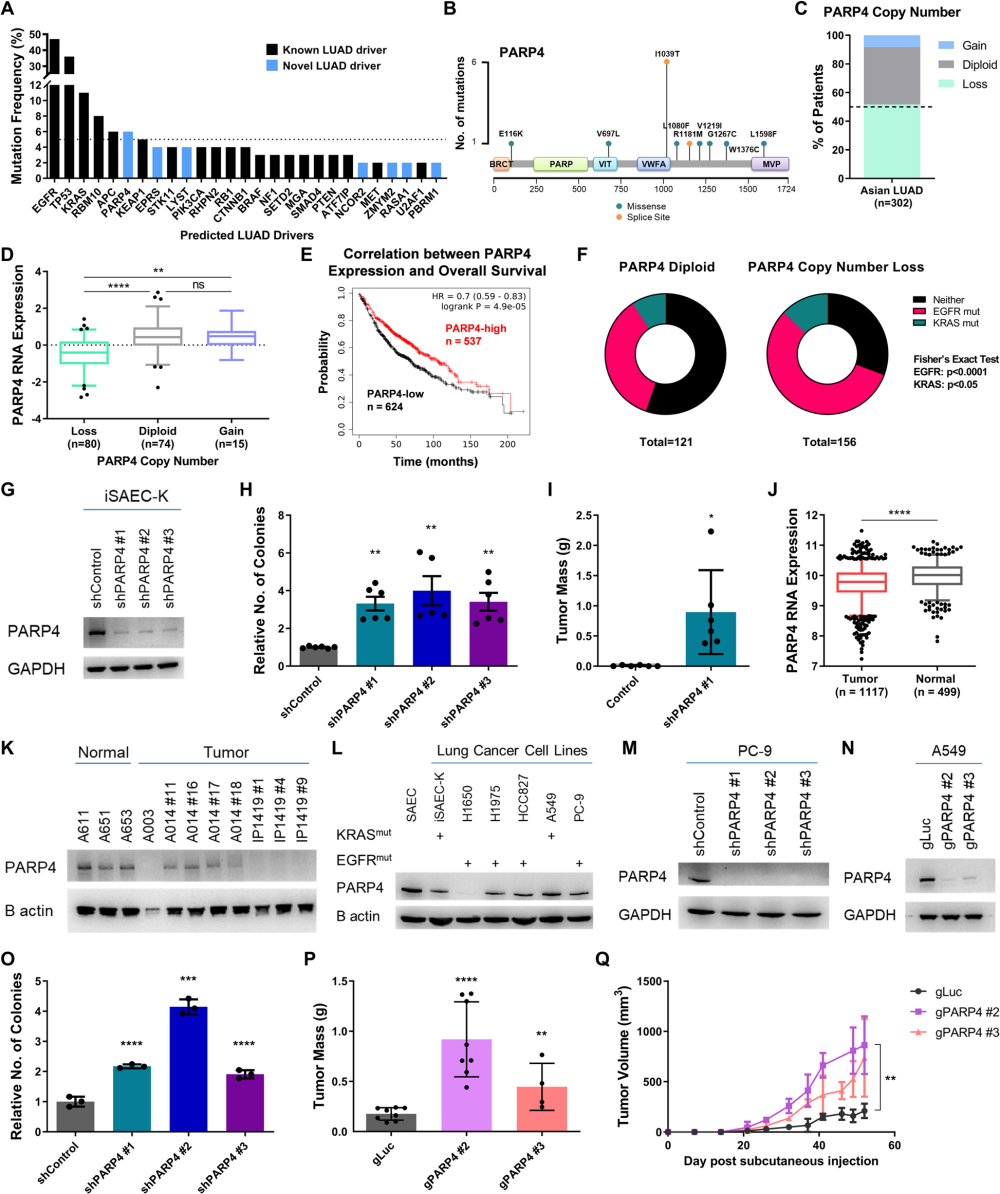
PARP4 is associated with tumor suppressive activity in LUAD. **A** Mutational frequency of driver genes identified from the Asian LUAD cohort [9]. **B** Distribution of non-silent PARP4 mutations from the Asian LUAD cohort. **C** Distribution of PARP4 copy number status within the Asian LUAD cohort. **D** PARP4 RNA expression z scores grouped by PARP4 copy number status in Asian LUAD patients. Boxes represent quartiles while whiskers extend to the 5th and 95th percentiles. **E** Kaplan–Meier plot generated using LUAD microarray data (*n* = 1161, Affymetrix ID 202239_at for PARP4) from the KM Plotter database, where data was aggregated from multiple cohorts across 12 GEO datasets [26, 27]. **F** Frequency of PARP4 diploid or copy number loss patients with a concurrent mutation in EGFR, KRAS or neither within the Asian LUAD cohort. **G** Immunoblot indicating reduction in expression of PARP4 upon shRNA knockdown in iSAEC-K cells. **H** Relative quantitation of soft agar colonies formed by iSAEC-K cells. Data represent the mean ± s.e.m., *n* ≥ 5. **I** Mass of tumors formed by iSAEC-K cells after 8 weeks. Data represent the mean ± s.d., *n* = 6. **J** PARP4 transcript levels in LUAD tumor (*n* = 1117) and normal tissues (*n* = 499). Boxes represent quartiles while whiskers extend to the 5th and 95th percentiles. Data were retrieved from the GENT2 database [28, 29]. **K** Immunoblot analysis of PARP4 expression in patient-derived lung cells. **L** Immunoblot analysis of PARP4 levels in a lung cell line panel, with KRAS and EGFR mutation status indicated [30]. **M** Immunoblot validation of PARP4 shRNA knockdown in PC-9 cells. **N** Immunoblot analysis of PARP4 expression following PARP4 pooled CRISPR knockout in A549 cells. **O** Relative quantitation of soft agar colonies formed by PC-9 cells. Data represent the mean ± s.e.m., *n* = 3. **P** Mass of tumors formed by A549 cells after 8 weeks. Data represent the mean ± s.d., *n* ≥ 4. **Q** Growth curve of tumors formed from A549 cells. Data represent the mean ± s.d., *n* ≥ 4

**Fig. 2:**
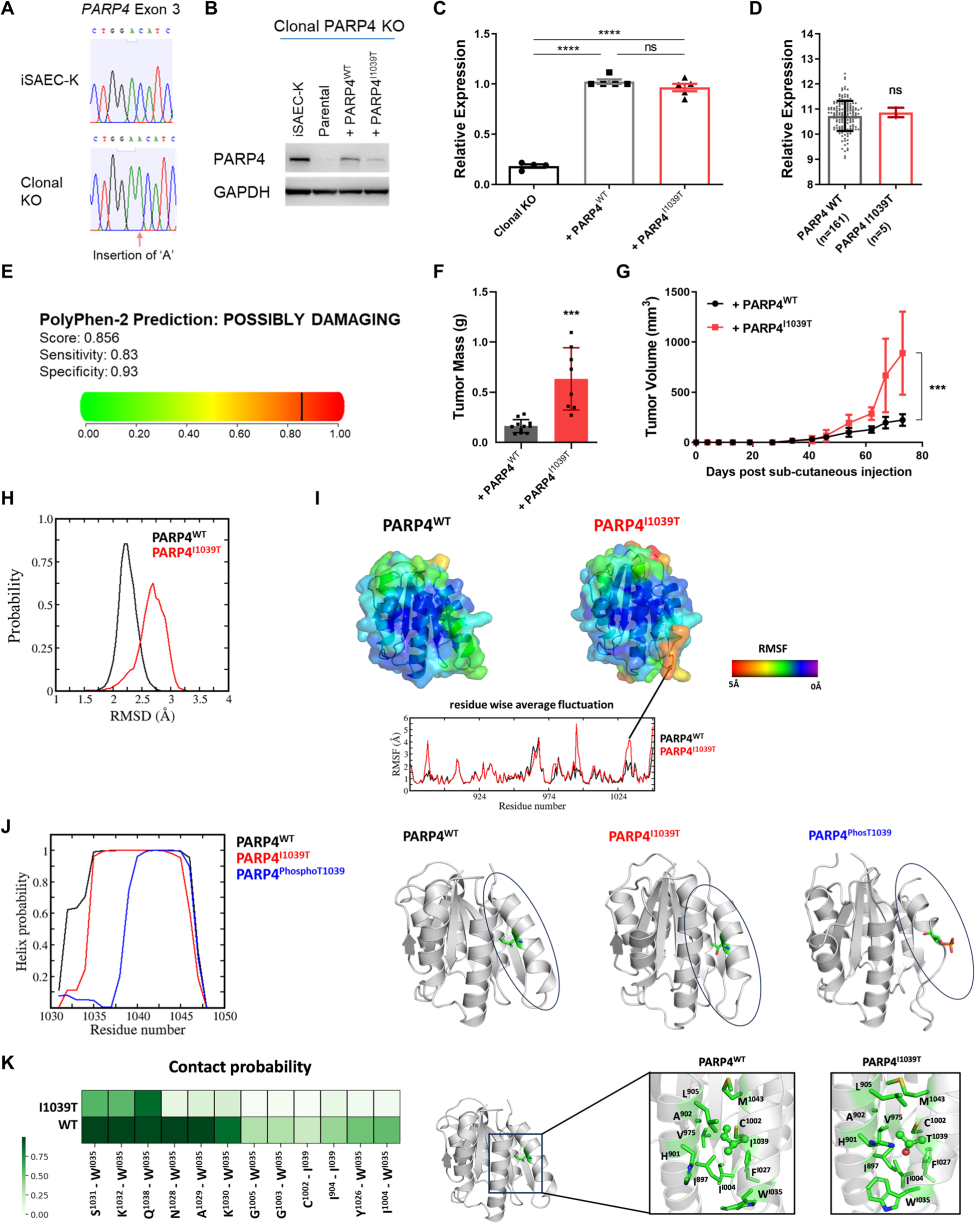
Recurrent I1039T mutation in PARP4 contributes to tumorigenicity. **A** Sanger sequencing chromatogram depicting frameshift mutation within PARP4 exon 3 in the PARP4 clonal KO iSAEC-K line. **B** Immunoblot analysis indicating lower PARP4 expression in clonal PARP4 KO cells overexpressing PARP4^WT^ compared to iSAEC-K cells, and even lower PARP4 expression in clonal PARP4 KO cells overexpressing mutant PARP4^I1039T^. **C** RT-qPCR analysis of PARP4 transcript levels. Data represent the mean ± s.e.m., *n* ≥ 4. **D** Relative PARP4 expression in PARP4^WT^ and PARP4^I1039T^ patients from the Asian LUAD cohort. Data represent the mean ± s.d., *n* ≥ 5. **E** Results from PolyPhen-2 analysis of the I1039T mutation [31]. **F** Mass of tumors formed by PARP4 clonal KO cells expressing PARP4^WT^ or PARP4^I1039T^ after 8 weeks. Data represent the mean ± s.d., *n* ≥ 8. **G** Growth curve of tumors in **F**. **H** Distribution of root mean square deviation of conformations sampled during molecular dynamics (MD) simulations of PARP4-VWFA^WT^ (black) and PARP4-VWFA^I1039T^ (red) against the initial PARP4-VWFA^WT^ model. **I** Residue-wise average root mean square fluctuation of all conformations sampled during the MD simulations of PARP4-VWFA^WT^ and PARP4-VWFA^I1039T^ mapped on to the corresponding structures. **J** Distribution of helical probability of residues from the alpha helix of PARP4-VWFA (left); MD snapshot of PARP4-VWFA with the I1039, T1039 and phosphorylated T1039 (PhosT1039) residues highlighted in stick representation (right). **K** Contact analysis highlighting the interactions between the residues from the alpha helix with surrounding residues in the PARP4-VWFA domain. A darker shade of green represents a higher contact probability (left); MD snapshot showing the orientation of residues surrounding residue 1039 in PARP4-VWFA^WT^ and PARP4-VWFA^I1039T^. I1039 and T1039 are shown in ball-and-stick representation while remaining residues are shown in stick representation (right)

Whole-exome sequencing [10] and RNA sequencing data [11] from patients of East Asian ancestry with lung adenocarcinoma from our previous cohort study [9] were used in this study. Full methodological details and characteristics of the cohort may be found in the accompanying paper for the study [9]. In brief, 213 lung adenocarcinoma patients of Chinese ethnicity were recruited from the National Cancer Center of Singapore with prior written informed consent. Tumor and adjacent normal tissues were collected following surgical resection and biopsy, respectively, and reviewed by pathologists to assess histological characteristics. Adjacent normal lung tissue or blood was used as a matched normal control. Paired whole exome sequencing (WES) was performed for 210 of these patients while RNA sequencing was conducted for 181 patients. The WES data was combined with that from 92 Chinese patients from an independently published study [12] for collective analysis through somatic variant identification, driver prediction, and copy number analysis pipelines. This dataset may be publicly accessed from the OncoSG portal without need for a coding interface [25]. Analyses for Fig. 1A, B, C, and F were conducted using all patient data where PARP4 copy number status was available (*n* = 302). For Fig. 1F, association between PARP4 copy number status and EGFR or KRAS mutation status was measured by Fisher’s exact test. Analyses for Figs. 1D and 2D were conducted using all patient data where PARP4 copy number and RNA expression data were concurrently available (*n* = 169). For the rMATS splicing analysis contributing to Fig. 6 (refer to the rMATS analysis section of the “Methods”), RNA data from PARP4 copy number loss patients with the lowest third of PARP4 expression levels (*n* = 27) were compared against that of PARP4 diploid patients with the highest third of PARP4 expression (*n* = 25). The full list of sample IDs used for each analysis can be found in Additional file 2.

### Cell lines and culture conditions

A549, H1975, HCC827, HEK293T, and Platinum-A (Plat-A) cell lines were maintained in Dulbecco's modified Eagle medium (DMEM) (Gibco) supplemented with 10% fetal bovine serum (FBS) (Gibco), 1% penicillin–streptomycin (Gibco), 2 mM L-glutamine (Gibco), and 1 mM sodium pyruvate (Gibco). H1650 and PC-9 cell lines were maintained in Roswell Park Memorial Institute 1640 medium (RPMI) (Gibco) supplemented with 10% FBS, 1% penicillin–streptomycin, 1% Minimum Essential Medium non-essential amino acids (MEM NEAA) (Gibco), 2 mM L-glutamine, and 1 mM sodium pyruvate. SAEC and patient-derived lung cells were maintained on irradiated NIH-3T3-J2 feeder layers in epithelial cell culture medium as previously described [32]. iSAEC and iSAEC-K cells were maintained in the same medium. Detailed protocols are described in Additional file 1: Methods.

### Soft agar assay

Two thousand five hundred cells were seeded in 1 mL of 0.35% (w/v) low-melt agarose (Bio-Rad) dissolved in DMEM/F12 medium supplemented with 10% FBS, 1% penicillin–streptomycin, 2 mM L-glutamine, and 1 mM sodium pyruvate (soft agar medium), atop a 1% (w/v) agarose layer in a 12-well. Of the respective cell line culture medium, 1 mL was added after agarose solidification. After 3 weeks, colonies were fixed in methanol and stained with crystal violet. Wells were imaged using a Gel Doc machine (Bio-Rad), and positively stained colonies visible by eye were counted.

### Tumor xenografts

All animal experiments were approved and carried out in accordance with the A*STAR Biological Resource Centre (A*STAR – BRC) Institutional Animal Care and Use Committee (IACUC) guidelines (Protocol number 171286). Animals were housed in a specific pathogen free facility with a 12-h light–dark cycle, under controlled temperature and humidity conditions. Animals had free access to sterile food and water. One million iSAEC-K cells or 400,000 A549 cells were resuspended in 100 µL media containing 50% Matrigel Matrix (Corning) and injected subcutaneously into both flanks of age-matched male or female NOD *scid* gamma (NSG) mice (The Jackson Laboratory) under isoflurane anesthesia. Mice were examined for tumor formation on a weekly basis, and palpable tumors were measured by Vernier caliper. Tumor volume was determined as follows: ½ × tumor length × (tumor width)^2^. After the tumors had formed, mice were monitored three times a week. After 8 to 10 weeks, with individual tumors not exceeding 15 mm in diameter, mice were euthanized by CO_2_ in a humane manner in accordance with the IACUC guidelines.

### Protein modeling

Structural models of WT—PARP4^VWFA^ (Additional file 3) and I1039T—PARP4^VWFA^ (Additional file 4) were generated using the program ColabFold [33], with the latter used to model the T1039 phosphorylated state, PhosT1039—PARP4^VWFA^. All generated models were refined using molecular dynamics (MD) simulations as we have done elsewhere [34]. The Xleap module of AMBER 18 [35] was used to prepare the systems for the MD simulations. Each simulation system was neutralized with appropriate numbers of counter ions and each neutralized system was solvated in an octahedral box with TIP3P [36] water molecules, leaving at least 10 Å between the solute atoms and the boundaries of the box. MD simulations were carried out with the pmemd.cuda module of the AMBER 18 package in combination with the ff14SB force field [37]. The parameters for phosphothreonine were as described elsewhere [38]. MD simulations were carried out in explicit solvent at 300 K. During the simulations, the long-range electrostatic interactions were treated with the particle mesh Ewald [39] method using a real space cut off distance of 9 Å. The settle [40] algorithm was used to constrain bond vibrations involving hydrogen atoms, which allowed a time step of 2 fs during the simulations. Solvent molecules and counter ions were initially relaxed using energy minimization with restraints on the protein atoms. This was followed by unrestrained energy minimization to remove any steric clashes. Subsequently the system was gradually heated from 0 to 300 K using MD simulations with positional restraints (force constant: 50 kcal mol^−1^ Å^−2^) on the protein atoms over a period of 0.25 ns allowing water molecules and ions to move freely followed by gradual removal of the positional restraints and a 2 ns unrestrained equilibration at 300 K. The resulting system was used as the starting structure for the production phase and three independent (using different initial random velocities) MD simulations were carried out for 100 ns. Accelerated MD simulations (aMD) with dual boost potential [41] were used to further enhance the conformational sampling of the simulated systems. Simulation trajectories were visualized using VMD [42] and figures were generated using Pymol [43].

### Immunoprecipitation

Cells were lysed in ice-cold immunoprecipitation lysis buffer (co-IP buffer) (10 mM Tris–HCl (pH 7.5), 150 mM NaCl, 0.5% (v/v) NP-40, 0.25% (w/v) sodium deoxycholate, and 0.5 mM EDTA (pH 8.0)) supplemented with 1X Halt™ Protease and Phosphatase Inhibitor Cocktail (Thermo Fisher Scientific), 2 mM MgCl_2,_ and 50 units/mL benzonase (Sigma-Aldrich) for 1 h and clarified by centrifugation at 21,000 × *g* for 20 min at 4 °C. Protein concentrations were determined using the Coomassie Plus™ Protein Assay Reagent (Thermo Fisher Scientific).

One milligram of total protein was pre-cleared for 1 h using 50 μL of ChIP-grade Protein A/G Dynabeads (Thermo Fisher Scientific) before overnight incubation with 4 μg of the immunoprecipitating antibody (Additional file 1: Table S11); 50 μL of fresh Protein A/G Dynabeads were blocked overnight in 1% (v/v) bovine serum albumin (BSA) (Hyclone) in co-IP buffer. The antibody-lysate mixtures were incubated with blocked Dynabeads for 90 min. Dynabeads were washed three times with co-IP buffer, and bound proteins were eluted in 2 × Laemmli buffer (Bio-Rad) at 95 °C for 5 min.

### Nanopore sequencing

Total RNA of the respective cell lines was extracted in duplicate using the RNeasy Mini Kit (Qiagen), with additional on-column DNA digestion. RNA quality was assessed using the Agilent 2100 Bioanalyzer Instrument (Agilent). Poly(A) RNA was enriched from 30 µg total RNA using the Dynabeads mRNA Purification Kit (Thermo Fisher Scientific). Nanopore direct cDNA libraries were generated from 500 ng of poly(A) RNA using the Direct cDNA kit, SQK-DCS109 (Oxford Nanopore Technologies). cDNA libraries were sequenced using the GridION device (release v20.10.6) from Oxford Nanopore Technologies, with one sample per GridION flowcell (FLO-MIN106D, R9.4.1). Samples were sequenced with a total run-time of 72 h. Live base-calling of reads was performed using Guppy 4.2.3 in high accuracy mode.

### Nanopore data analysis

Base-called reads with quality scores ≥ 7 were aligned using Minimap2 (2.17-r941) to the GRCh38 reference human genome. PSI-Sigma (v1.9) was used to identify alternative splicing events with at least five supporting reads. A PSI value was calculated to measure the inclusion rate of each splice event. A splicing event was considered significantly dysregulated between the experimental and control duplicates if changes in > 10% PSI and *p* value < 0.05 were measured.

### rMATS analysis

Splicing analysis for single exon skipping (SES) and intron retention (IR) events was performed on short-read RNA sequencing data [9, 11] using rMATS (version 4.1.0, using default parameters). Splicing in PARP4 copy number loss patients with the lowest third of PARP4 expression levels were compared against PARP4 diploid patients with the highest third of PARP4 expression (Additional file 2). Events with |inclusion level difference|> 0.05 and *p* value < 0.05 were identified as significantly dysregulated splicing events. We drew reference from work by several others in the field, where the |inclusion level difference|> 0.05 threshold was frequently used and widely accepted [44–49].

### PCR validation of splice events

PCR amplification of cDNA samples was performed using Phusion HF Master Mix (Thermo Fisher Scientific) with the primers in Additional file 1: Table S12. PCR products were separated by gel electrophoresis (2% agarose in TBE buffer), imaged on the Gel Doc system (Bio-Rad) and quantitated using Image Lab software (Bio-Rad).

### Statistical analyses

Statistical analyses were performed using GraphPad Prism. Two-tailed unpaired Student’s *t* test was used to evaluate the significance of differences between two sample groups. For comparisons between multiple groups, ordinary one-way ANOVA was followed by either the Tukey test to correct for multiple comparisons between groups or the Dunnett test to correct for multiple comparisons to a control group. The multiplicity adjusted *p* value was then reported. A *p* value of less than 0.05 was defined as statistically significant. Significance values are indicated as follows: ns, not significant *p* ≥ 0.05, *p < 0.05, **p < 0.01, ***p < 0.001, and ****p < 0.0001.

## Results

### Functional genomics identifies PARP4 as a novel tumor suppressor in lung adenocarcinoma

Prior analysis of the Asian LUAD cohort data [9, 10] revealed seven frequently mutated genes (PARP4 (6%), EPRS (4%), LYST (4%), NCOR2 (2%), ZMYM2 (2%), RASA1 (2%), and PBRM1 (2%)) that had not been functionally characterized in LUAD (Fig. 1A). Of these, PARP4 was mutated at the highest frequency, placing it among the ranks of other well-known LUAD drivers such as KEAP1 (5%) and STK11 (4%). Furthermore, we noted a recurrent I1039T mutation shared by one-third of the 17 PARP4 mutant cases [10] (Fig. 1B), suggesting a functional significance that has not been previously elucidated. Strikingly, we observed multiple modes of putative dysregulation in PARP4 expression or function. Apart from 6% of the Asian LUAD patients bearing PARP4 mutations, the majority of patients within the Asian LUAD cohort had PARP4 copy number deletion that was accompanied by reduced PARP4 mRNA expression [10, 11] (Fig. 1C, D). Notably, when overall survival was compared between low and high PARP4-expressing LUAD patients (*n* = 1161) from publicly available datasets [26, 27], lower PARP4 expression levels were correlated with significantly poorer overall survival (Fig. 1E). Additionally, mutations in EGFR (47%) and KRAS (11%), which are the major mutually exclusive oncogenic drivers in the LUAD cohort [10], were significantly associated with PARP4 copy number loss (Fig. 1F, Additional file 1: Figure S1A). This suggested a previously unanticipated modulatory role of PARP4 in relation to oncogene-driven carcinogenesis and led us to examine the consequence of its loss in the context of oncogenic mutations.

We wanted to study PARP4 function in a suitable cell line model with a genetically defined background. There has been much interest in pinpointing the cell of origin of lung cancer, with different regions of the lung having been observed to promote the formation of different lung cancer subtypes [50]. Altogether, these results suggest that LUAD can arise from different progenitor cell populations depending on the microenvironmental context and oncogenic driver [51, 52]. As there is some consensus that LUAD arises from the distal lung regions [53, 54], we chose to use small airway epithelial cells (SAEC) originating from these distal regions as a model system for our experiments, although we acknowledge that they may not fully represent the entire spectrum of LUAD origins. Primary SAEC cells were first immortalized by overexpression of TERT and SV40 large T antigen (SV40LT) to form iSAEC [50]. Following immortalization, iSAEC was further transformed by overexpression of constitutively active KRAS^G12V^ (iSAEC-K) (Additional file 1: Figure S1B-D) [55]. Only with the expression of all three genetic elements were the iSAEC-K cells able to form soft agar colonies, as well as tumor xenografts in immunodeficient NSG mice (Additional file 1: Figure S1E). In contrast, iSAEC overexpressing TERT and SV40LT were unable to do so (Additional file 1: Figure S1E).

Upon shRNA knockdown of PARP4 in iSAEC-K (Fig. 1G), there was no significant difference in 2D proliferation (Additional file 1: Figure S1F). Interestingly, however, shPARP4 cells consistently formed a greater number of soft agar colonies (Fig. 1H), indicating their heightened ability for anchorage-independent growth. Furthermore, they formed substantially larger tumors compared to control (Fig. 1I), thereby demonstrating enhanced in vivo tumorigenicity upon PARP4 loss. This increase in tumorigenicity was similarly observed using an orthogonal method of depleting PARP4 through gRNA-mediated CRISPR knockout (Additional file 1: Figure S1G-I). Examination of publicly available lung cancer gene expression datasets [28, 29] revealed that PARP4 expression levels were lower in lung cancer samples than in matched normal tissue (Fig. 1J); this underscored the observation that PARP4 expression is downregulated or lost during lung cancer progression. In a panel of patient-derived lung normal and tumor cell lines that we established, PARP4 protein levels were indeed lower in tumor cell lines (Fig. 1K), further supporting PARP4’s putative tumor suppressive function.

As PARP4 copy number loss was associated with EGFR and KRAS mutations, we selected two additional classic lung cancer cell lines bearing EGFR or KRAS mutations that had moderate basal PARP4 expression (Fig. 1L). PC-9 has a deletion of amino acids 746 to 750 (exon 19 deletion) that renders EGFR constitutively active whereas A549 harbors constitutively-active KRAS^G12S^ [30]. PC-9 cells bearing shPARP4 knockdown were able to form a significantly larger number of soft agar colonies (Fig. 1M, O). Similarly, PARP4-depleted A549 cells formed larger tumors (Fig. 1N, P, Q). The observations underscored the broader relevance of PARP4’s tumor suppressive activity beyond our genetically-defined iSAEC-K cellular model. In the non-tumorigenic iSAEC, however, PARP4 knockdown cells remained unable to form soft agar colonies (Additional file 1: Figure S1J-L), indicating that PARP4 loss alone is insufficient for tumorigenesis and requires an additional oncogenic insult. This finding is consistent with the earlier analysis of the Asian LUAD genomics dataset [10] suggesting an association between the occurrence of EGFR or KRAS mutations and PARP4 copy number loss (Fig. 1F).

Quite strikingly, we noted that the recurrent PARP4 I1039T mutation observed in the Asian LUAD cohort was also seen in other cancer cohorts [56, 57] at low frequencies (Additional file 1: Table S1). Until now, this mutation has not been annotated as a mutation of significance on the Cancer Mutation Census curated by the Catalogue of Somatic Mutations in Cancer (COSMIC) database [58], and its functional relevance remains to be clarified. To assess the consequence of the I1039T mutation in cancer, we compared the in vivo tumor-forming ability of iSAEC-K cells overexpressing either PARP4^I1039T^ or PARP4^WT^ in a clonal PARP4 knockout background (Fig. 2A, B). Despite comparable PARP4 overexpression at the transcript level between both cell lines (Fig. 2C), PARP4^I1039T^ mutant cells consistently had lower PARP4 protein levels than the wild-type control cells (Fig. 2B). In fact, there was no significant difference in PARP4 transcript levels between I1039T mutant and wild-type PARP4 cases in the Asian LUAD cohort [10, 11] (Fig. 2D), indicating that the I1039T mutation does not alter PARP4 transcript levels in either the endogenous context or in our overexpression cell line system. We further examined the possible functional outcome of the I1039T mutation using the PolyPhen-2 tool [31], which predicts the impact of amino acid substitutions on protein structure and function. The PARP4 I1039T mutation had a probability score of 0.856 and was predicted to be “possibly damaging” (Fig. 2E), suggesting a potentially deleterious effect leading to loss of PARP4 function. Similar to what was observed upon PARP4 loss, the mutant I1039T line formed larger tumors than their wild-type counterpart (Fig. 2F, G).

To predict the structural effect of the I1039T mutation on PARP4 stability, we performed protein modeling experiments. As there are no known experimental structures of full-length multi-domain PARP4 protein, ColabFold [33] was used to construct a model of wild-type and mutant VWFA protein domain where the I1039 residue is located. We performed molecular dynamics simulations on these models and show that the structure of the mutant PARP4^I1039T^ deviated from that of PARP4^WT^, as seen from the higher root mean square deviation (RMSD) value (Fig. 2H). There was also greater fluctuation in the PARP4 mutant VWFA domain (Fig. 2I) particularly in the region of the mutation. The mutant residue T1039 in PARP4^I1039T^ lies in an alpha helix that undergoes destabilization in the mutant (Fig. 2J), where several contacts originally present between the helix and the surrounding VWFA domain were lost (Fig. 2K). This further strengthens the suggestion that the mutation could destabilize the VWFA structure. In addition, it has been predicted that the I1039T mutation is a potential phosphorylation site (NetPhos 3.1, probability score 0.889) [59], and our molecular dynamics simulations of phosphorylated T1039 (PARP4^phosT1039^) revealed even greater destabilization of the helix compared to the unphosphorylated mutant state (Fig. 2J). These findings support the notion that I1039T is a loss-of-function mutation that destabilizes PARP4 and functionally contributes to tumorigenicity.

### PARP4 mediates its tumor-suppressive function independently of the vault complex

**Fig. 3:**
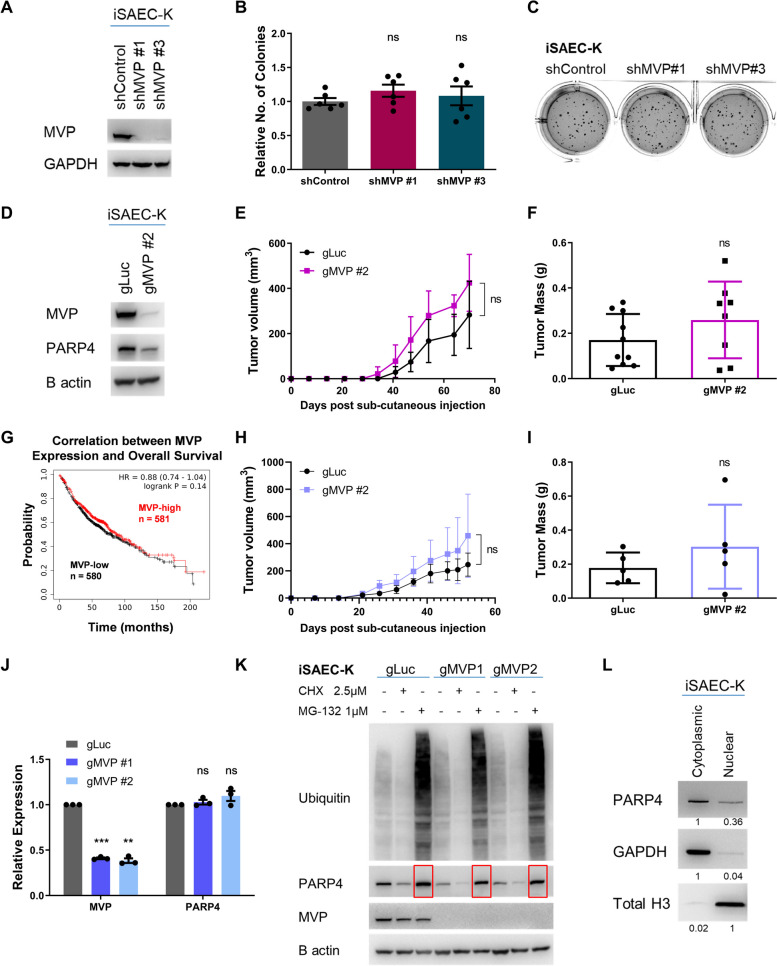
PARP4’s tumor suppressive activity is independent of the vault complex. **A** Immunoblot validation of MVP knockdown in iSAEC-K cells. **B** Relative quantitation of soft agar colonies. Data represent the mean ± s.e.m., *n* = 6. **C** Representative images of soft agar colonies stained by crystal violet. **D** Immunoblot validation of MVP depletion via pooled CRISPR knockout in iSAEC-K cells. **E** Growth curve of tumors formed from iSAEC-K cells. Data represent the mean ± s.d., *n* ≥ 8. **F** Mass of iSAEC-K tumors harvested after 9 weeks. Data represent the mean ± s.d., *n* ≥ 8. **G** Kaplan–Meier plot generated using LUAD microarray data (*n* = 1161, Affymetrix ID 202180_s_at for MVP) from the KM Plotter database, where data was aggregated from multiple cohorts across 12 GEO datasets [26, 27]. **H** Growth curve of tumors formed from A549 cells. Data represent the mean ± s.d., *n* = 5. **I** Mass of A549 tumors harvested after 7 weeks. Data represent the mean ± s.d., *n* = 5. **J** RT-qPCR analysis of MVP and PARP4 transcript levels in iSAEC-K gMVP #1 and gMVP #2 cells relative to gLuc. Data represent the mean ± s.e.m., *n* = 3. **K** Immunoblot analysis of PARP4 protein levels in control and MVP-depleted iSAEC-K cells at steady state, upon cycloheximide (CHX) inhibition of protein synthesis, or MG-132 inhibition of proteasomal degradation. Cells were treated with the indicated concentrations of CHX and MG-132 for 24 h. Red boxes indicate increased PARP4 protein following MG-132 treatment. **L** Immunoblot comparing PARP4 protein levels between the cytoplasmic and nuclear fraction of iSAEC-K cells. Equal amounts of total protein were used. GAPDH and total histone H3 were respectively used as cytoplasmic- and nuclear-specific markers. Band intensity was quantified relative to the cytoplasmic or nuclear fraction and indicated

Given the established role of PARP4 as a key subunit within the vault complex, we proceeded to determine if the vault complex is responsible for tumor-suppression. To do so, we depleted MVP, which is the sole component responsible for the structural integrity of the vault complex [19, 60], and assessed the resultant effect on tumorigenicity. ShRNA knockdown (Fig. 3A) or gRNA knockout (Fig. 3D) of MVP led to a slight downregulation of PARP4, consistent with previous reports suggesting that MVP stabilizes PARP4 by binding to PARP4’s C-terminal interaction domain and facilitating its incorporation within the vault complex [61, 62]. Surprisingly, however, MVP loss did not phenocopy our previous observations on PARP4 loss, as there was no significant difference in the number of soft agar colonies formed (Fig. 3B, C) or the size of tumor xenografts (Fig. 3E, F, H, I). Unlike PARP4, MVP expression levels had no significant impact on overall patient survival [26, 27] (Fig. 3G), indicating a surprising and unanticipated vault-independent function of PARP4 in cancer.

To determine the regulatory mechanisms between MVP and PARP4 stability, we first examined the effect of MVP loss on PARP4 transcript levels but found no significant difference (Fig. 3J), supporting prior observations that MVP affected PARP4 expression at the post-transcriptional level. We subsequently exposed control and MVP knockout iSAEC-K and A549 cells to inhibitors of protein synthesis and proteasomal degradation to assess PARP4 protein stability. Treatment with the proteasomal inhibitor MG-132 expectedly led to accumulation of ubiquitinated proteins that were not cleared by the proteasome (Fig. 3K). In MVP-depleted iSAEC-K and A549 cells, we observed the accumulation and stabilization of PARP4 levels following MG-132 treatment, indicating that the stability of a significant fraction of PARP4 protein indeed depended on MVP (Fig. 3K, red boxes, Additional file 1: Figure S2A, B). However, under steady state untreated conditions, PARP4 expression was not completely abolished in the MVP-depleted cells. This suggests that some PARP4 protein could exist independently of MVP and appeared resistant to MVP loss. Consistent with our observations, previous reports have also highlighted non-vault-associated forms of PARP4 [14, 18, 19, 63]. As PARP4 possesses predicted nuclear localization sequences and has been previously observed in the nuclear matrix, we performed cellular fractionation experiments on iSAEC-K cells and indeed observed a subset of PARP4 residing in the nuclear fraction (Fig. 3L), where vault complexes were known to be excluded due to their size [14, 18, 64]. These findings collectively indicated possible vault-independent roles for PARP4, prompting us to explore novel PARP4 binding partners that could contribute to PARP4’s anti-tumor functionalities.

### hnRNPM is a PARP4 binding partner that phenocopies the function of PARP4

**Fig. 4:**
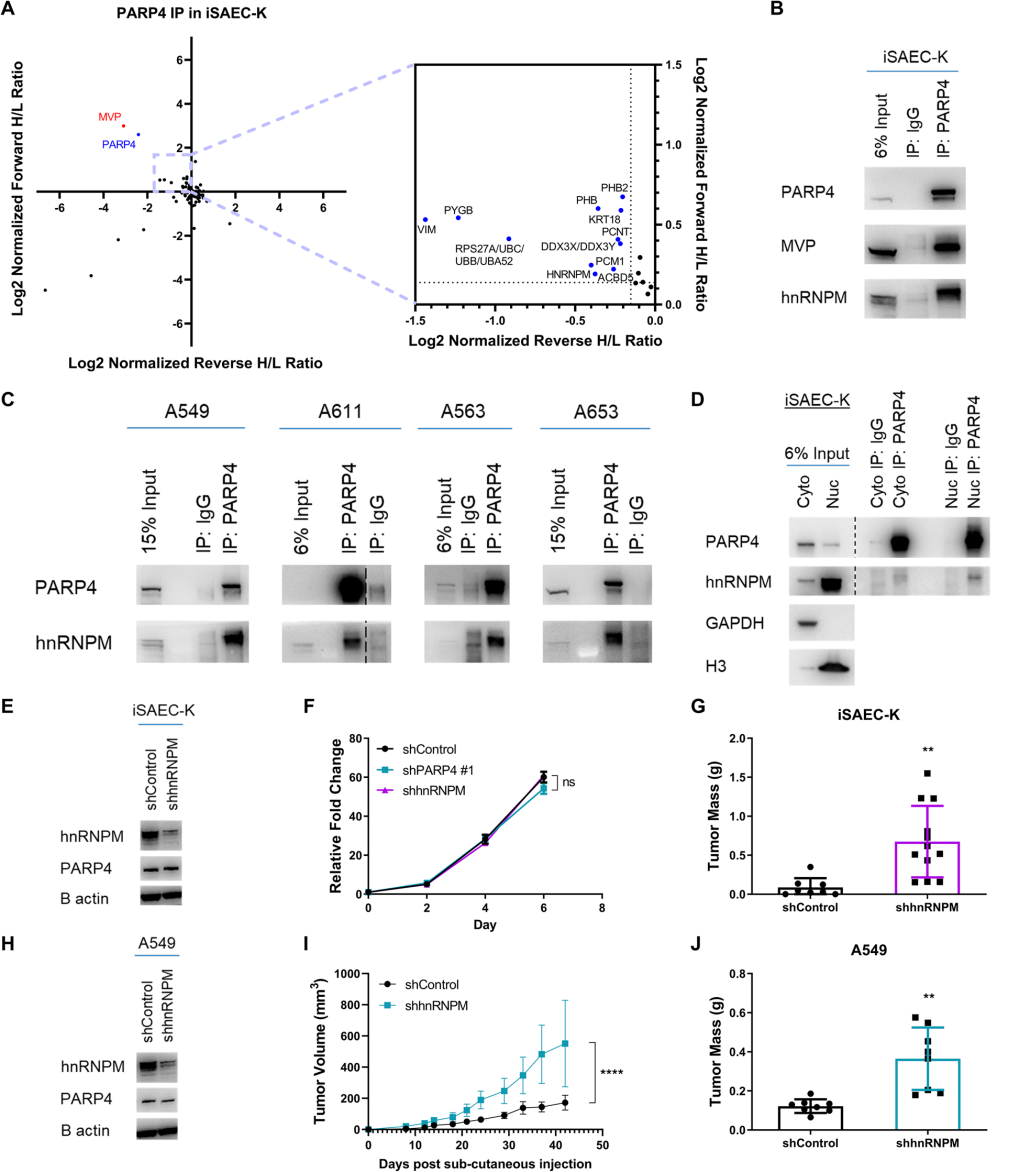
hnRNPM is a potentially novel PARP4 binding partner with tumor suppressive activity in LUAD. **A** Log_2_ normalized forward and reverse SILAC Heavy/Light (H/L) ratios of proteins detected in the PARP4 SILAC co-IP mass spectrometry experiment. Candidate proteins with forward H/L ratio > 1.1 and reverse H/L ratio < 0.9 are found in the top left quadrant. The bait protein PARP4 is labeled in blue while PARP4’s known interaction partner MVP is labeled in red (left); enlarged plot area for better resolution of candidate proteins (right). **B** Immunoblot analysis following immunoprecipitation of PARP4 in iSAEC-K cells. **C** Immunoblot analysis following immunoprecipitation of PARP4 in the lung cell lines A549, A611, A563 and A653. **D** Immunoblot analysis following immunoprecipitation of PARP4 from the cytoplasmic (cyto) and nuclear (nuc) fractions of iSAEC-K cells. GAPDH was used as a cytoplasmic marker while total histone H3 was used as a nuclear marker. **E** Immunoblot analysis of hnRNPM and PARP4 levels in iSAEC-K shControl and shhnRNPM cells. **F** Proliferative capacity of iSAEC-K shControl, shPARP4 #1 and shhnRNPM cells as measured by the CellTiter-Glo assay. Data represent the mean ± s.e.m., *n* = 3. **G** Mass of tumors formed from iSAEC-K shControl or shhnRNPM cells after 10 weeks. Data represent the mean ± s.d., *n* ≥ 8. **H** Immunoblot analysis of hnRNPM and PARP4 levels in A549 shControl and shhnRNPM cells. **I** Growth curve of tumors formed from A549 shControl or shhnRNPM cells. Data represent the mean ± s.d., *n* = 8. **J** Mass of tumors in **I**

To systematically identify endogenous interactors of PARP4, we performed PARP4 immunoprecipitation using stable isotope labelling by amino acids in cell culture (SILAC) labeled cell extracts and subjected them to quantitative mass spectrometry analysis [65]. Immunoprecipitation of PARP4 was performed in iSAEC-K shControl and shPARP4 #1 cells, with the latter serving as a negative control to improve the specificity of the binding partners identified. The bait protein PARP4 and its main interacting partner MVP were the most highly enriched proteins (Fig. 4A). In the SILAC setup, specific interaction partners possessed inverse ratios between the forward and reverse experiments and thus clustered in the upper-left quadrant. Interestingly, several proteins that were not previously known to interact with PARP4, such as heterogeneous ribonucleoprotein M (hnRNPM), were identified in this quadrant, with forward SILAC ratio > 1.1 and reverse SILAC ratio < 0.9) (Additional file 1: Table S2).

To validate the candidate interaction partners, we performed immunoprecipitation of either PARP4 or its candidate partners in iSAEC-K lysates, thus allowing for the detection of native, endogenous interactions. Immunoprecipitation of PARP4 resulted in the co-immunoprecipitation of not only MVP, as expected, but also hnRNPM (Fig. 4B). In addition, we found that targeted immunoprecipitation of pericentrin (PCNT), pericentriolar material 1 protein (PCM1), keratin 18 (KRT18), and vimentin (VIM) co-immunoprecipitated PARP4 (Additional file 1: Figure S3A-C). Among the candidate interaction partners, hnRNPM consistently co-immunoprecipitated with PARP4 in multiple lung cell lines including A549 and several patient-derived cell lines (Fig. 4C), thereby compelling further investigations into the nature and functional significance of this interaction.

hnRNPM is a predominantly nuclear protein, although it has been reported to shuttle between the cytoplasm and nucleus [66, 67]. Given our previous observation that PARP4 exists in the nucleus of iSAEC-K cells, we next sought to determine the localization of PARP4’s interaction with hnRNPM. Immunoprecipitation of PARP4 performed on cytoplasmic and nuclear fractions of iSAEC-K cells revealed hnRNPM to be co-immunoprecipitated predominantly in the nuclear fraction (Fig. 4D). PARP4 did not control hnRNPM localization, as immunofluorescence showed no distinct changes to hnRNPM’s nuclear staining upon PARP4 loss (Additional file 1: Figure S3D). Depletion of PARP4 in iSAEC-K and A549 cells did not significantly affect steady state protein levels of hnRNPM either (Additional file 1: Figure S3E). This observation was supported by proteomic data from 106 lung adenocarcinoma patient tumors [68, 69], which revealed no significant correlation between PARP4 and hnRNPM protein expression (Additional file 1: Figure S3F).

We thus examined the involvement of PARP4’s ADP-ribosylation catalytic activity in its tumor suppressive function by overexpressing mutant PARP4 lacking its PARP catalytic domain in PARP4 clonal knockout iSAEC-K cells, which were used in subsequent in vivo tumorigenicity assays (Additional file 1: Figure S3G, H). Compared to wild-type PARP4, the PARP domain deletion resulted in larger tumors (Additional file 1: Figure S3I, J), signifying the importance of the PARP domain to PARP4’s tumor suppressive activity. By immunoprecipitating PARP4 in iSAEC-K cells and probing the eluates for mono-ADP-ribose (MAR) modifications, we observed the presence of unique bands that were absent in the IgG negative control (Additional file 1: Figure S3K, yellow arrows), suggesting that PARP4 could be responsible for modifications on its binding partners. Notably, in line with previous in vitro radiolabelling experiments where PARP4 was observed to ADP-ribosylate itself as well as MVP [14, 70], bands corresponding to the molecular weight of PARP4 (193 kDa) and MVP (100 kDa) were observed. Interestingly, a band corresponding to the expected molecular weight of hnRNPM (72–75 kDa) was also observed in the PARP4 eluate that was absent in the negative IgG control, raising the possibility that hnRNPM could be an ADP-ribosylation target of PARP4.

Functionally, hnRNPM belongs to the family of heterogeneous ribonucleoproteins (hnRNPs), which comprises RNA-binding proteins integral to various aspects of RNA regulation, such as in regulating splicing and mRNA stability [67]. Post-translational modification, including ADP-ribosylation, of hnRNPs, has been shown to alter their splicing activity [71–73]. In the context of cancer, hnRNPM has been reported to drive a number of tumor-supporting splicing programs or events [74–76]. However, the role of hnRNPM in the context of lung cancer and its regulation by PARP4 has not been clarified. Similar to PARP4, lower expression of hnRNPM was correlated with poorer overall survival from the same publicly available LUAD microarray datasets [26, 27] (Additional file 1: Figure S3L). Depletion of hnRNPM in iSAEC-K and A549 cells using shRNAs revealed that while there was no significant difference in cell proliferation rates (Fig. 4E, F, H, Additional file 1: Figure S3M), hnRNPM knockdown cells formed significantly larger tumors upon subcutaneous implantation into the flanks of immunocompromised NSG mice (Fig. 4G, I, J). To some degree, hnRNPM loss recapitulated the phenotype of PARP4 loss in vivo, leading us to hypothesize that hnRNPM could also be an important downstream regulator of LUAD tumorigenicity.

To identify PARP4-hnRNPM interaction site(s), we chose the HEK293T cell line which expresses hnRNPM but has low expression of PARP4 compared to the A549 and iSAEC-K lung cancer cells (Additional file 1: Figure S4A). This enables us to assess the interaction of exogenously introduced FLAG-tagged full-length or fragments of PARP4 with hnRNPM (Additional file 1: Figure S4B). Across replicates, strongest enrichment of hnRNPM was observed from FLAG immunoprecipitation of PARP4-Fragment 3 (amino acid residues 1115–1724) and to a smaller degree with PARP4-Fragment 2 (amino acid residues 623–1114). In contrast, the interaction was not observed with PARP4-Fragment 1 (amino acid residues 1–622) (Additional file 1: Figure S4C). Deletion of the VIT or VWFA domains located in PARP4-Fragment 2, or the MVP interaction domain within PARP4-Fragment 3, did not ablate PARP4’s interaction with hnRNPM (Additional file 1: Figure S4D), suggesting that the intervening sequences between the domains (amino acid residues 744–883 between VIT and VWFA, amino acid residues 1055–1569 between VWFA and MVP) may be key to this interaction. To assess if PARP-hnRNPM interaction is essential for the tumor suppressive function of PARP4, we overexpressed PARP4 fragments in PARP4 clonal knockout A549 cells (Additional file 1: Figure S4E, F). Overexpression of PARP4-Fragment 3 resulted in significantly fewer soft agar colonies than empty vector- or PARP4-Fragment 2-expressing cells (Additional file 1: Figure S4G, H), suggesting that PARP4-hnRNPM interaction is important in reducing tumorigenicity. Interestingly, PARP4-Fragment 1-expressing cells also had significantly reduced soft agar colony forming ability, indicating the contribution of PARP4-Fragment 1 to PARP4’s tumor suppressive functionality, possibly through its ADP-ribosylation activity (Additional file 1: Figure S3G-K). It would be interesting to further examine the relative contributions of PARP4 protein regions and integration with its enzymatic activity to PARP4’s tumor suppressive function.

### Perturbations to hnRNPM and PARP4 disrupt splicing events

Having demonstrated hnRNPM as a prospective new association partner of PARP4, we sought to examine the underlying mechanisms by which it exerts its effects in cancer. Since hnRNPM is a splicing regulatory factor with a majority of studies focused on its RNA-binding properties and effects on splicing outcomes [74, 77], we examined hnRNPM-dependent splicing activity in lung cancer. To understand the impact of splicing mediated by hnRNPM, as well as the potential role of PARP4 in regulating this process, we performed Nanopore sequencing in iSAEC-K shControl, shhnRNPM, and shPARP4#1 cell lines [78] to detect the range of splicing alterations resulting from the loss of either hnRNPM or PARP4. Using the PSI-Sigma pipeline, which was selected for its capability in detecting and quantitating alternative splicing events using long-read sequencing data [79], we examined five types of alternative splicing events: (1) single exon skipping (SES), (2) multiple exon skipping (MES), (3) intron retention (IR), and use of either (4) an alternative 5’ splice site (A5SS), or (5) an alternative 3’ splice site (A3SS) at a particular exon (Additional file 1: Figure S5A).

**Fig. 5:**
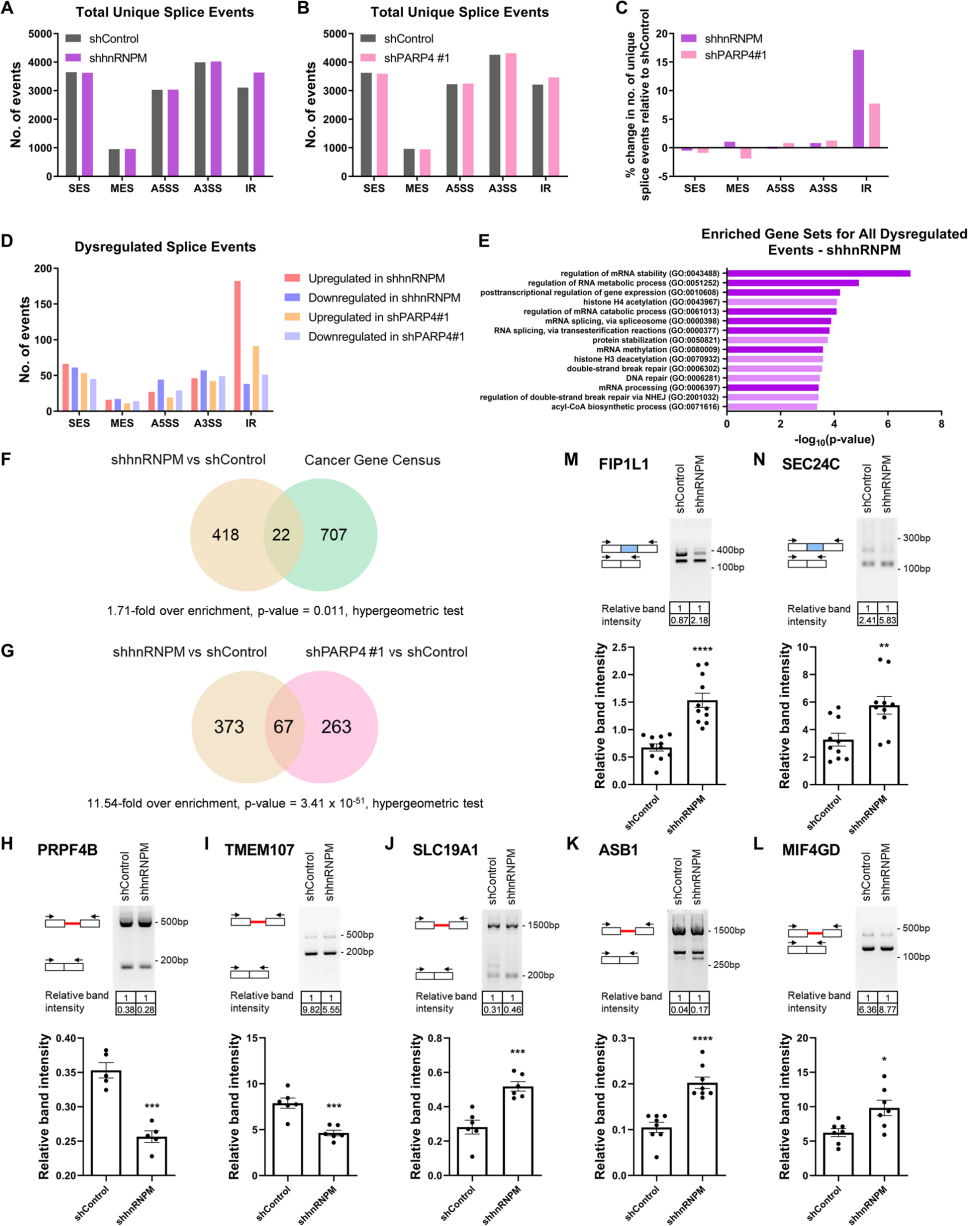
hnRNPM and PARP4 regulate splicing in the LUAD context. **A, B** Number of unique splice events with ≥ 5 supporting sequencing reads detected across the five event categories in **A**) iSAEC-K shControl and shhnRNPM cells and **B**) iSAEC-K shControl and shPARP4#1 cells. **C** Percentage change in number of unique splice events detected across the five event categories in iSAEC-K shhnRNPM (purple) or shPARP4#1 (pink) relative to shControl cells. **D** Number of significantly upregulated or downregulated splice events (|ΔPSI|> 10, *p* value < 0.05) upon hnRNPM or PARP4 loss across the five event categories. **E** Top 15 enriched GO Biological Process 2021 gene sets among genes with significantly dysregulated splicing upon hnRNPM knockdown (|ΔPSI|> 10, *p* value < 0.05). Gene sets related to RNA metabolism and splicing are highlighted with a darker shade. **F** Overlap between genes with splicing regulated by hnRNPM and cancer-related genes defined by COSMIC [58], with 1.71-fold over enrichment and *p* value = 0.011, as determined by hypergeometric test. **G** Overlap between genes with splicing regulated by hnRNPM and genes with splicing regulated by PARP4, with 11.54-fold over enrichment and *p* value = 3.41 × 10^–51^, as determined by hypergeometric test. **H**-**N** Representative gel electrophoresis images of targeted PCR validation of upregulated (**H, I**) and downregulated (**J, K, L**) IR events, as well as (**M, N**) upregulated SES events in iSAEC-K shControl and shhnRNPM samples. Band intensity was quantified, with that of the lower band normalized to that of the upper band, and indicated below the respective lanes. To the left of the respective bands, a schematic diagram indicates the splicing outcome. The red line represents the retained intron, the blue bar represents the alternatively skipped exon, while black arrows represent the PCR primers. The bar graph at the bottom summarizes the results from experimental replicates. Data represent the mean ± s.e.m., *n* ≥ 5

From our analysis, splice events across each of the five event categories were detected in control, hnRNPM-, and PARP4-knockdown iSAEC-K cells. In all three cell lines, the greatest number of events belonged to the A3SS category, with over 4000 unique events detected, while MES events were least frequent, with close to 1000 unique events detected (Fig. 5A, B). More importantly, 17% more unique intron retention events were detected in the hnRNPM knockdown cells than in the control cells, whereas the other categories of splice events had similar unique event counts between the two conditions (Fig. 5C). The same was true for PARP4 knockdown cells, although the effect was more modest, with there being 8% more unique intron retention events detected. Furthermore, in both comparisons, intron retention events were also the most dysregulated class of splicing events, with the majority of these being significantly upregulated (ΔPSI > 10, *p* value < 0.05) (Fig. 5D). This provided the first indication that hnRNPM and PARP4 likely promoted intron removal from certain genes in lung cancer, but the precise mechanism remains to be determined. Single exon skipping events were the next most dysregulated class of events. In the case of hnRNPM, the numbers of up- (ΔPSI < − 10) and down-regulated (ΔPSI > 10) single exon skipping events were comparable—66 and 61, respectively (Fig. 5D), suggesting that hnRNPM promotes both exon inclusion and exclusion events in iSAEC-K cells. This finding is consistent with the recently reported role of hnRNPM in mediating exon inclusion and exclusion, in contrast to the classical notion of hnRNPs as splicing repressors [76, 77].

To gather a broad perspective of splicing programs affected in hnRNPM knockdown cells, gene ontology (GO) enrichment analysis was performed on the set of genes with significantly dysregulated splicing events. RNA processing functions were most highly enriched (Fig. 5E), consistent with hnRNPM’s role in regulating its own splicing and that of other RNA-binding and splicing-related proteins. Furthermore, among the 729 COSMIC cancer genes [58], the splicing of 22 genes was regulated by hnRNPM. This corresponded to 5% of all alternatively spliced genes upon hnRNPM loss (Fig. 5F, 1.71-fold over enrichment, *p* value = 0.011, hypergeometric test), indicating that hnRNPM was likely involved in the splicing of known cancer-associated genes. In the case of PARP4 knockdown, RNA transport processes were also enriched (Additional file 1: Figure S5B). There was a significant overlap of 67 genes that were alternatively spliced upon PARP4 loss (Fig. 5G, 11.54-fold over enrichment, *p* value = 3.41 × 10^–51^, hypergeometric test); 17% of genes with dysregulated IR events upon PARP4 loss were shared with hnRNPM (Additional file 1: Figure S5C, 35.51-fold over enrichment, *p* value = 1.59 × 10^–19^, hypergeometric test), while for SES events, this overlap was 15.4% (Additional file 1: Figure S5D, 11.54-fold over enrichment, *p* value = 31.79 × 10^–17^, hypergeometric test). This indicated that PARP4 knockdown shared a number of commonalities in splicing outcomes with hnRNPM knockdown.

We next selected the top dysregulated IR and SES events identified (Additional file 1: Table S3-6) for targeted PCR validation. Specifically, primers were designed to flank the target region to amplify alternative forms of the transcript, thereby yielding PCR products with different sizes that could be separated by gel electrophoresis. We first focused on validating the top hnRNPM-regulated events as splicing alterations were more pronounced upon hnRNPM loss. We were able to confirm the expected changes in dysregulated IR and SES events in hnRNPM knockdown cells. Upon hnRNPM loss, there was increased intron retention for PRPF4B—a kinase that also has roles in splicing [80], as well as TMEM107, which is involved in Sonic hedgehog signaling [81] (Fig. 5H, I). hnRNPM loss also led to decreased intron retention for SLC19A1—a folate transporter [82], ASB1—implicated in proteasomal degradation and inflammation [83], as well as MIF4GD, which has roles in cell cycle regulation [84] (Fig. 5J–L). In the case of SES events, hnRNPM knockdown resulted in increased exon skipping in both FIP1L1, which is one of the 22 overlapping COSMIC genes and is involved in mRNA polyadenylation [85], and SEC24C, which is a coat protein on vesicles from the endoplasmic reticulum that is involved in the sorting and transport of cargo [86] (Fig. 5M, N). Whereas the functional significance of these validated splicing events has not been previously reported, they were predicted to alter the splicing outcome between a functional transcript isoform versus one that has no protein product (Additional file 1: Figure S5E-K).

### Dysregulation of splicing observed in Asian LUAD cohort cases with PARP4 copy number loss

**Fig. 6:**
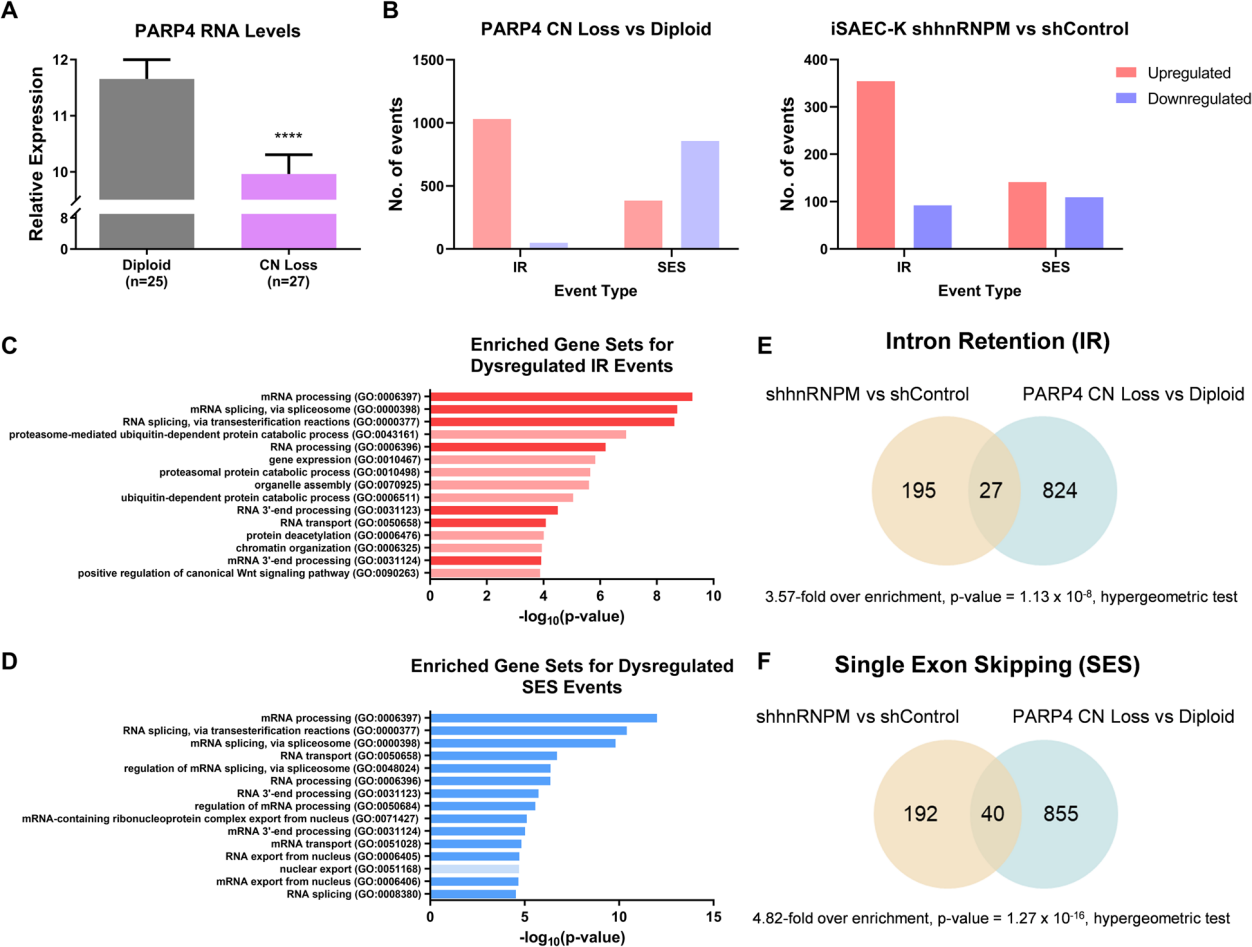
Dysregulation of splicing observed in Asian LUAD cohort cases with PARP4 copy number loss. **A** PARP4 RNA expression in PARP4 diploid versus PARP4 copy number loss cases stratified by PARP4 expression levels. Data represent the mean ± s.d. **B** Number of significantly upregulated and downregulated IR and SES events (|ΔPSI|> 5 and *p* value < 0.05) detected by rMATS analysis of stratified PARP4 copy number loss versus diploid patients [11] (left) or PSI-Sigma analysis of iSAEC-K shhnRNPM versus shControl cells [78] (right). **C, D** Top 15 enriched GO Biological Process 2021 gene sets among genes with significantly dysregulated **C**) IR or **D**) SES events in the PARP4 copy number loss versus PARP4 diploid splicing analysis (|ΔPSI|> 5, *p* value < 0.05). Gene sets related to RNA metabolism and splicing are highlighted with a darker shade. **E**, **F** Overlap in genes with significantly dysregulated (|ΔPSI|> 5, *p* value < 0.05) **E**) IR or **F**) SES identified from the iSAEC-K shhnRNPM versus shControl analysis and the PARP4 copy number loss versus diploid analysis, with **E**) 3.57-fold over enrichment and *p* value = 1.13 × 10^–8^, or **F**) 4.82-fold over enrichment and *p* value = 1.27 × 10^–16^, as determined by hypergeometric test

As PARP4 is a key interaction partner of hnRNPM, with cell line data suggesting that PARP4 could affect splicing events [78], we sought to determine whether splicing was similarly perturbed by changes in PARP4 levels in clinical lung samples. We first examined RNA-seq data from the Asian LUAD cohort [11] to compare splicing profiles between patient tumors with PARP4 copy number loss and those with diploid PARP4 [10]. This was to obtain an indication of how PARP4 contributes to the splicing landscape in LUAD, as well as to identify clinically relevant splice events. We identified diploid PARP4 cases with high PARP4 expression levels (*n* = 25) and compared these with copy number loss cases with low PARP4 expression levels (*n* = 27) (Fig. 6A, Additional file 2). Our analysis revealed a total of 1030 significantly upregulated and 49 significantly downregulated IR events, as well as 383 significantly upregulated and 857 significantly downregulated SES events (Fig. 6B). The greater number of upregulated versus downregulated IR events mirrored the experimental loss of hnRNPM in cells (Fig. 6B), strongly correlating with the role of PARP4 in regulating hnRNPM. Similar to the case of hnRNPM, these events were collectively enriched in genes involved in RNA regulation, with splicing, RNA processing, and RNA export among the top significantly and highly enriched gene sets (Fig. 6C, D). More strikingly, a subset of genes with dysregulated IR and SES events was shared between the cell line and patient data splicing analyses. Specifically, 12% of genes with hnRNPM-regulated IR events (Fig. 6E, 3.57-fold over enrichment, *p* value = 1.13 × 10^–8^, hypergeometric test) and 17% of genes with hnRNPM-regulated SES events (Fig. 6F, 4.82-fold over enrichment, *p* value = 1.27 × 10^–16^, hypergeometric test) were also differentially spliced between the PARP4 copy number loss and diploid groups. Taken together, these findings provide insights into how the loss of PARP4 function in LUAD could regulate splicing events, possibly through hnRNPM, thereby contributing to lung cancer pathogenesis.

## Discussion

PARP4 is among the commonly mutated genes associated with LUAD tumorigenesis, but its functional relevance has not been well-elucidated. Prior to this study, PARP4 knockout mice were found to be more likely than wild-type mice to develop colon tumors when challenged with the chemical carcinogen dimethylhydrazine, underscoring its potential tumor suppressive role [20]. PARP4 has also been proposed as a candidate cancer susceptibility gene in thyroid and breast cancers [23, 24], suggesting PARP4’s involvement in other cancer types. However, depletion of PARP4 in various cell line models yielded contrasting in vitro effects, including both enhanced and reduced proliferation [23, 24, 87]. In our study, loss of PARP4 led to increased tumorigenicity despite lacking observable changes in proliferation rates.

The recurrent I1039T mutation in PARP4 identified in the Asian LUAD cohort was predicted to be deleterious and shown to be more tumorigenic. Given the predicted deleterious effect, as well as the lower protein levels despite matched RNA levels, we speculated that the I1039T mutation might destabilize the protein, thereby reducing PARP4 levels and resulting effectively in a loss-of-function phenotype. Deleterious amino acid substitutions are known to replace key functional residues, such as those involved in catalytic activity or post-translational modification, or affect protein scaffolding by destabilizing interaction sites [88, 89]. In the case of the I1039T mutation, isoleucine, a nonpolar amino acid, is converted to threonine, a polar amino acid bearing a hydroxyl group that is amenable to post-translation modifications. In fact, the T1039 residue was predicted with high likelihood as a potential phosphorylation site, and as we show from our protein modeling data, whether phosphorylated or unphosphorylated, the presence of either a negative charge or polar group where there was originally a hydrophobic residue could disrupt protein interactions important in stabilizing PARP4.

While PARP4 has been reported in some instances to exist outside the vault complex [14, 18, 63, 87], the precise functions for these vault-independent fractions remain to be established. Here, we provide the first evidence of a vault-independent tumor suppressive role for PARP4. We have identified hnRNPM as a potential novel PARP4 interaction partner and perturbed splicing, with a propensity for upregulated intron retention events, as a point of convergence of PARP4 and hnRNPM loss. We raise the possibility of PARP4 binding to and ADP-ribosylating hnRNPM, as post-translational modification of hnRNPM has previously been shown to alter its splicing activity. In one study, the phosphorylation status of specific serine residues within hnRNPM’s RNA recognition motifs directly affected hnRNPM’s splicing regulation in mouse macrophages [71]. Intriguingly, a search in the ADPriboDB 2.0 database [90, 91] of known ADP-ribosylation modifications showed that hnRNPM could be ADP-ribosylated by PARP1, although the exact site and function of this modification were not reported. Furthermore, ADP-ribosylation of other hnRNPs had previously been shown to modulate splicing [72, 73]. As there has not been any global profiling study defining PARP4’s ADP-ribosylation targets, unlike what has been done for several of the PARP family members [90–94], it could be informative to systematically identify PARP4’s ADP-ribosylation targets via mass spectrometry.

Interestingly, hnRNPM has mostly been reported to drive tumor-promoting splicing programs in several cancer types. These include splicing events that promote the epithelial to mesenchymal transition to support metastasis in breast and gastric cancer [76, 95], as well as events that limit the efficacy of chemotherapeutic agents, thereby having a protective effect in Ewing sarcoma [75, 96]. However, we and others observe a contrasting tumor-suppressive role for hnRNPM in our respective experimental contexts of lung cancer and prostate cancer [97]. This duality is not surprising, as splicing factors such as RBM5 and ESPR1/2 have been found to have contrasting roles in different cancer contexts [98]. The hnRNP family is also no exception, with hnRNPK being a classic example with seemingly dichotomous roles in tumorigenesis [99], serving as a transcriptional coactivator for p53 on one hand [100, 101] and stimulating the expression of c-Myc to drive proliferation on the other [102, 103]. Taken together, we propose that the pleiotropic activities of these splicing factors may lead to oncogenic or tumor-suppressive outcomes in different disease contexts.

Dysregulated splicing has been increasingly recognized to underlie the cancer phenotype [104]. At the global level across cancer types, an increased diversity of alternative splicing events, including novel splice junctions and widespread intron retention, has been detected uniquely in patient tumors compared to normal samples and these are thought to diversify the tumor transcriptome [105]. These splicing changes can be attributed to mutations in splicing regulatory *cis* elements of cancer-associated genes, as well as changes to the activity and expression levels of core and auxiliary splicing factors. For example, mutations in the splicing regulator RBM10 promote exon 9 inclusion in the Notch signaling inhibitor *numb* transcript, giving rise to an alternative protein isoform that stimulates Notch pathway activation and proliferation in lung cancer [106]. Other instances of specific splicing alterations have been found to modulate the different hallmarks of cancer [107]. This study provides evidence to suggest a genetic link wherein changes to PARP4 copy number cascade to altered hnRNPM splicing and reveal potential splicing events perturbed upon PARP4 or hnRNPM loss. This warrants further efforts to examine the functional significance of splice events identified from our study.

## Conclusions

Using the largest Asian LUAD patient dataset as a starting point, we have identified PARP4 copy number loss or mutation at relatively high frequency, which we subsequently validated in vitro and in vivo as an important modulator of tumor progression. While previous studies of PARP4 have largely been limited to its association with the vault complex, we discovered a striking vault-independent role for PARP4 in suppressing tumorigenicity. Instead, we established hnRNPM as a novel PARP4 interaction partner that is also key to regulating LUAD tumorigenicity. Having observed commonalities in patterns of splicing alterations across PARP4 and hnRNPM loss, we hypothesize that perturbations to PARP4 or hnRNPM result in dysregulated splicing underlying LUAD tumorigenicity. This work contributes towards the precision medicine effort by identifying additional modulators of tumorigenicity that are clinically relevant and represents a step in addressing the growing need to bridge genetic mutations identified from patient tumor sequencing studies with their functional relevance. Beyond the existing focus on well-known driver genes, we show that there is room to explore other players that regulate tumor progression, and this could broaden the repertoire of therapeutic targets and biomarkers in lung adenocarcinoma.

### Supplementary Information

**Additional file 1:** Supplementary Information. PDF file containing additional methods, Supplementary figures S1-S5 with corresponding figure legends, and Supplementary tables S1-S12.

**Additional file 2.** List of Asian LUAD sample IDs. Excel file containing the list of patient cohort sample IDs used in the analyses for the respective figures.

**Additional file 3. **ColabFold model of WT - PARP4^VWFA^. PDB file containing the structural model of the WT - PARP4^VWFA^ domain generated using ColabFold. File can be viewed using the RCSB Protein Data Bank web viewer (https://www.rcsb.org/3d-view).

**Additional file 4. **ColabFold model of I1039T - PARP4^VWFA^. PDB file containing the structural model of the I1039T - PARP4^VWFA^ domain generated using ColabFold. File can be viewed using the RCSB Protein Data Bank web viewer (https://www.rcsb.org/3d-view).

## Data Availability

Raw mass spectrometry data for this study were generated at the Quantitative Proteomics Core (Cancer Science Institute of Singapore). The mass spectrometry proteomics data have been deposited to the ProteomeXchange Consortium via the PRIDE [108] partner repository with the dataset identifier PXD050844 [65]. Raw Nanopore sequencing data for this study were generated at the Integrated Genomics Platform (Genome Institute of Singapore) and has been deposited on the Sequence Read Archive (SRA) under BioProject ID PRJNA1066997 [78]. Raw whole-exome sequencing data and RNA sequencing data from the Asian lung adenocarcinoma cohort [9] analyzed in this study had been deposited on the European Genome-phenome Archive (EGA) under accession codes EGAD00001004422 [10] and EGAD00001004421 [11], respectively, and may also be accessed from the OncoSG data portal under dataset “Lung Adenocarcinoma (NCCS/GIS, 2020)” [25]. PDB files for the initial structural models of WT—PARP4^VWFA^ and I1039T—PARP4^VWFA^, on which further MD simulations were performed as described in the “Methods” section, are provided in Additional files 3 and 4. The computational models will be made available upon request.

## Abbreviations

A3SS: Alternative 3’ splice site
A5SS: Alternative 5’ splice site
CN: Copy number
COSMIC: Catalogue Of Somatic Mutations in Cancer
Co-IP: Co-immunoprecipitation
GO: Gene ontology
IR: Intron retention
iSAEC: Immortalized small airway epithelial cells
iSAEC-K: Immortalized small airway epithelial cells overexpressing KRAS^G12V^
LUAD: Lung adenocarcinoma
MD: Molecular dynamics
MES: Multiple exon skipping
NSG: NOD *scid* gamma
PSI: Percent spliced in
SAEC: Small airway epithelial cells
SES: Single exon skipping
SILAC: Stable isotope labelling by amino acids in cell culture
