# Supplementary material for: PARP4 interacts with hnRNPM to regulate splicing during lung cancer progression
Source: Genome Med. 2024 Jul 22;16:91. doi: 10.1186/s13073-024-01328-1 (PMC11265163; doi:10.1186/s13073-024-01328-1)
Supplement: Supplementary file 1 — Additional file 1: Supplementary Information. PDF file containing additional methods, Supplementary figures S1-S5 with corresponding figure legends, and Supplementary tables S1-S12. [file 13073_2024_1328_MOESM1_ESM.pdf]

Supplementary Information for

PARP4 interacts with hnRNPM to regulate splicing during lung cancer progression

Yi Fei Lee[1,2], Cheryl Zi Jin Phua[1], Ju Yuan[1], Bin Zhang[3,4,5], May Yin Lee[1], Srinivasaraghavan Kannan[6], Yui Hei Jasper Chiu[1], Casslynn Wei Qian Koh[1], Choon Kong Yap[1], Edwin Kok Hao Lim[1], Jianbin Chen[1], Yuhua Lim[1], Jane Jia Hui Lee[1], Anders Jacobsen Skanderup[1], Zhenxun Wang[1,7], Weiwei Zhai[1,8,9], Nguan Soon Tan[2,10], Chandra S. Verma[2,6,11], Yvonne Tay[3,12,13], Daniel Shao Weng Tan[14], Wai Leong Tam[1,3,12,13]

[1] Genome Institute of Singapore, Agency for Science, Technology and Research, Singapore.
[2] School of Biological Sciences, Nanyang Technological University, Singapore.
[3] Cancer Science Institute of Singapore, National University of Singapore, Singapore
[4] Computational Bioscience Research Center, King Abdullah University of Science and Technology (KAUST), Thuwal, Saudi Arabia.
[5] Computer Science Program, Computer, Electrical and Mathematical Sciences and Engineering Division, King Abdullah University of Science and Technology (KAUST), Thuwal, Saudi Arabia
[6] Bioinformatics Institute, Agency for Science, Technology and Research, Singapore. Singapore
[7] Centre for Vision Research, Duke-NUS Medical School, Singapore
[8] Key Laboratory of Zoological Systematics and Evolution, Institute of Zoology, Chinese Academy of Sciences, Beijing, China.
[9] Center for Excellence in Animal Evolution and Genetics, Chinese Academy of Sciences, Kunming, China.
[10] Lee Kong Chian School of Medicine, Nanyang Technological University, Singapore.
[11] Department of Biological Sciences, National University of Singapore, Singapore
[12] NUS Centre for Cancer Research, Yong Loo Lin School of Medicine, National University of Singapore, Singapore
[13] Department of Biochemistry, Yong Loo Lin School of Medicine, National University of Singapore, Singapore
[14] Division of Medical Oncology, National Cancer Centre Singapore, Singapore.

Corresponding author: Tam Wai Leong
Email: tamwl@gis.a-star.edu.sg

This PDF file includes:

Additional methods
Figures S1 to S5
Tables S1 to S12
References

Methods

Generation of iSAEC and iSAEC-K

iSAEC cells were generated by the sequential transduction of SAEC cells (Lonza Catalog No. CC-2547) with retroviral supernatant generated from the following plasmids: pBABE-puro SV40 LT (Addgene #13970) and pBABE-hygro-hTERT (Addgene #1773). Cells were selected with puromycin and hygromycin and taken off the feeder layers. iSAEC-K cells were generated by the further transduction of iSAEC with retroviral supernatant generated from the pBABE puro K-Ras V12 (Addgene #9052) plasmid.

Generation of patient-derived cell lines

Patient tissue was processed following collection by patient-consented surgery (Agency for Science, Technology and Research, Singapore (A*STAR) IRB no. 2020-149). Tumor or adjacent normal tissue samples were minced finely using a scalpel, followed by enzymatic dissociation using collagenase IV and dispase II at 37°C for 1 hour. Cell suspensions were rinsed and passed through 100 µm cell strainers (BD Falcon) before plating on irradiated NIH-3T3-J2 feeder layers in epithelial cell culture medium described above.

Cell proliferation assay

500-1,000 cells were seeded in triplicate in a total volume of 100 µL per well, on 96-well Greiner flat-bottomed white plates (Sigma-Aldrich). Cell viability was measured using the CellTiter-Glo Luminescent Cell Viability Assay (Promega) at 24- or 48-hour intervals over a duration of 4 to 6 days.

Cloning

pLKO-EGFP was generated by replacing the puromycin cassette within pLKO.1 puro (Addgene #8453) with the EGFP cassette from LV-GFP (Addgene #25999). Briefly, both plasmids were digested using BamHI and KpnI restriction enzymes (New England Biolabs). The linearized pLKO.1 puro backbone and EGFP cassette were gel-extracted using the Qiagen Gel Extraction Kit and ligated using the Thermo Fisher Scientific Rapid Ligation Kit. The shRNA sequences in Supplementary Table 7 were subsequently cloned into pLKO-EGFP linearized by MluI and EcoRI (New England Biolabs). The gRNA sequences in Supplementary Table 8 were cloned into LentiCRISPRv2GFP (Addgene #82416) linearized by BsmBI (New England Biolabs). PARP4 open reading frame (ORF) was purchased from Bio Basic Asia Pacific Pte Ltd and subcloned to replace the EF1a-eFFly cassette of the pCDH-EF1a-eFFly-mCherry plasmid (Addgene #104833) to generate pCDH-PARP4-T2A-mCherry. PCR-based site-directed mutagenesis to generate the I1039T mutation and truncated PARP4 variants was performed using the KAPA HiFi PCR Kit (Roche) with the following primer pairs:

I1039T Forward – 5’ AACAGACAGAAGACCAAATGACCAGGCTATGTTCTC 3’;
I1039T Reverse – 5’ GGTCTTCTGTCTGTTTTCTCCAACTATGCTTGGATTTTGC 3’;
PARPdel Forward – 5’ ACCCAACTTTCATCCTAGTGATCATACTGAATTAGAGG 3’;
PARPdel Reverse – 5’ GATGAAAGTTGGGTTTGGACAAATTAGTTTCACAGAC 3’;
VITdel Forward – 5’ AACTTCCGAACTCAGCATCCTGGGCA 3’;
VITdel Reverse – 5’ TGAGTTCGGAAGTTTTGGCATCTGGTAACTGG 3’;
VWFAdel Forward – 5’ TGAGAGCTGTTCTCCGAGTTGCCACTC 3’;
VWFAdel Reverse – 5’ GAGAACAGCTCTCACTGGCTAGGTCAGG 3’;
MVPdel Forward – 5’ ACACTGGCTCCATTACAGTCAAGGCGC 3’;
MVPdel Reverse – 5’ AATGGAGCCAGTGTTGTATGCACACTATTTCATCCTC 3’

The NEBuilder HiFi DNA Assembly Master Mix (New England Biolabs) was used according to manufacturer’s instructions to clone Fragments 1-3 of PARP4 into the vector construct with the following primer pairs:

Vector Forward – 5’ GCGGCCGCTGAGGGCAGA 3’;
Vector Reverse – 5’ CTTGTCATCGTCGTCCTTGTAGTCCATGGTG 3’;
Fragment 1 Forward – 5’ ACAAGGACGACGATGACAAGGTGATGGGAATCTTTGCAAATTG 3’;

Fragment 1 Reverse – 5' CCTCTGCCCTCAGCGGCCGCAGGAACCAAGTTCCCAGAG 3';
Fragment 2 Forward – 5' ACAAGGACGACGATGACAAGCTGGAGGATGTCCACATC 3';
Fragment 2 Reverse – 5' CCTCTGCCCTCAGCGGCCGCAGTAGTCGACACCATTGTAC 3';
Fragment 3 Forward – 5' ACAAGGACGACGATGACAAGGAGCTTCAGAAGACAACTG 3';
Fragment 3 Reverse – 5' CCTCTGCCCTCAGCGGCCGCGCCTTGACTGTAATGGAG 3';

Transfection and generation of stable cell lines
HEK293T cells were transfected with the respective lentiviral constructs as well as the pCMV-dR8.2 dvpr (Addgene #8455) and pCMV-VSV-G (Addgene #8454) packaging plasmids using the FuGENE 6 Transfection Reagent (Promega) for lentivirus production. Plat-A cells were transfected with the respective retroviral constructs alongside the pUMVC (Addgene #8449) and pCMV-VSV-G (Addgene #8454) packaging plasmids in the same manner. Viral supernatant was collected 48 hours post-transfection and filtered before use. Cells were transduced with the viral supernatant using polybrene (Sigma-Aldrich) at a final concentration of 8 ng/µL.

Generation of clonal PARP4 knockout lines
iSAEC-K cells were first transduced with gPARP4 #2 or gPARP4 #3. FACS sorting of single GFP+ cells into each well of several 96-well plates was performed. Single colonies were expanded, followed by protein extraction and immunoblotting to check for loss of PARP4. Genomic DNA was then extracted from successful clones using the DNeasy Blood and Tissue Kit (Qiagen). PCR amplification of PARP4 exon 3 was performed using Phusion HF Master Mix (Thermo Fisher Scientific) with the following primer pair: Forward – 5' ACCTTTGAACCTCCCTTTCCA 3'; Reverse – 5' ACCCAAGCGTGTACTATGGC 3'. PCR products were sent for Sanger sequencing to check for homozygous frameshift mutations at the gRNA target site.

RNA isolation and quantitation via Real Time Quantitative Polymerase Chain Reaction (RT–qPCR)
RNA extraction was performed using the RNeasy Mini Kit (Qiagen) in conjunction with TRIzol® Reagent (Thermo Fisher Scientific). 500 µg of total RNA was converted to cDNA using the High-Capacity cDNA Reverse Transcription Kit (Applied Biosystems) and diluted fivefold. Transcript levels were measured with gene-specific primers (Supplementary Table 9) using the BlitzAmp Hotstart qPCR Master Mix (MiRXES) and QuantStudio™ 5 Real-Time PCR System (Applied Biosystems). Results were normalized to GAPDH.

Protein extraction
Cells were lysed in ice-cold RIPA Lysis and Extraction Buffer (Thermo Fisher Scientific) supplemented with 1X Halt™ Protease and Phosphatase Inhibitor Cocktail (Thermo Fisher Scientific) on ice for 30 minutes. Sonication was performed for 5 cycles of 30 s ON and 30 s OFF at high setting on the Bioruptor Plus (Diagenode). Lysates were clarified by centrifugation at 21,000 x g for 20 minutes at 4°C. Protein concentrations were determined using the Coomassie Plus™ Protein Assay Reagent (Thermo Fisher Scientific).

Cell fractionation
Cell fractionation was performed using the PARIS™ kit (Thermo Fisher Scientific) according to the manufacturer's protocol. The cytoplasmic and nuclear fractions were clarified by centrifugation at 21,000 x g for 20 minutes at 4°C. Protein concentrations were determined using the Coomassie Plus™ Protein Assay Reagent (Thermo Fisher Scientific).

Immunoblotting
30-50 µg of protein lysates were heated with NuPAGE™ LDS Sample Buffer and Sample Reducing Agent (Thermo Fisher Scientific) at 70°C for 10 minutes. Samples were resolved on 4-12% NuPAGE™ Bis-Tris precast gels (Thermo Fisher Scientific) and transferred onto PVDF membranes (Trans-Blot® Turbo™ Transfer System, Bio-Rad). Membranes were blocked in 5% (w/v) milk (Bio-Rad) in Tris-buffered saline containing 0.1% Tween-20 (TBS-T) and incubated in primary antibody at 4°C overnight. Membranes were incubated with a horseradish peroxidase–conjugated secondary antibody at room temperature for an hour. Protein bands were visualized using the

SuperSignal West Dura Extended Duration Substrate (Thermo Fisher Scientific) and ChemiDoc MP Imaging System (Bio-Rad). Antibody details are described in Supplementary Table 10.

Stable Isotope Labeling by Amino Acids in Cell Culture (SILAC)

Cells were incubated in DMEM (-Arg, -Lys) containing 10% dialyzed fetal bovine serum (Thermo Fisher Scientific) supplemented with 42 mg/l $^{13}C_6{}^{15}N_4$ L-arginine and 73 mg/l $^{13}C_6{}^{15}N_2$ L-lysine (Cambridge Isotope) or the corresponding non-labeled amino acids. Successful SILAC incorporation was verified by in-gel trypsin digestion and mass spectrometry analysis of heavy input samples to ensure an incorporation rate of >98%.

Mass spectrometry analysis

Samples were heated at 95°C prior to separation on a 12% NuPAGE™ Bis-Tris precast gel (Thermo Fisher Scientific) for 10 min at 170 V in 1x MOPS buffer, followed by gel fixation using the Colloidal Blue Staining Kit (Thermo Fisher Scientific). For in-gel digestion, samples were destained in destaining buffer (25 mM ammonium bicarbonate; 50% ethanol), reduced in 10 mM DTT for 1 h at 56°C followed by alkylation with 55 mM iodoacetamide (Sigma-Aldrich) for 45 min in the dark. Tryptic digestion was performed in 50 mM ammonium bicarbonate buffer with 2 µg trypsin (Promega) at 37°C overnight. Peptides were desalted on StageTips and analyzed by nanoflow liquid chromatography on an EASY-nLC 1200 system coupled to a Q Exactive HF mass spectrometer (Thermo Fisher Scientific). Peptides were separated on a C18-reversed phase column (25 cm long, 75 µm inner diameter) packed in-house with ReproSil-Pur C18-AQ 1.9 µm resin (Dr Maisch). The column was mounted on an Easy Flex Nano Source, and the temperature was controlled by a column oven (Sonation) at 40°C. A 105-min gradient from 2 to 40% acetonitrile in 0.5% formic acid at a flow of 225 nl/min was used. Spray voltage was set to 2.2 kV. The Q Exactive HF was operated with a TOP20 MS/MS spectra acquisition method per MS full scan. MS scans were conducted with 60,000 at a maximum injection time of 20 ms and MS/MS scans with 15,000 resolution at a maximum injection time of 50 ms. The raw files were processed with MaxQuant [1] version 1.5.2.8 and searched against the human UniProt database (95,934 entries) with preset standard settings for SILAC labeled samples, and the re-quantify option was activated. Carbamidomethylation was set as a fixed modification, while methionine oxidation and protein N-acetylation were considered variable modifications. Search results were filtered with a false discovery rate of 0.01. Known contaminants, proteins groups only identified by site, and reverse hits of the MaxQuant results were removed, and only proteins were kept that were quantified by SILAC ratios in both 'forward' and 'reverse' samples. The mass spectrometry proteomics data have been deposited to the ProteomeXchange Consortium via the PRIDE [2] partner repository with the dataset identifier PXD050844 [3].

FLAG tag immunoprecipitation

12.5 µL of ChromoTek DYKDDDDK Fab-Trap™ Agarose (Proteintech) bead slurry was incubated with 500 µg of co-immunoprecipitation lysate, and FLAG immunoprecipitation was performed following manufacturer's instructions. In brief, each sample was topped up to a total volume of 400 µL using dilution buffer. Samples were incubated for 90 min on rotation at 4°C before washing and elution in 2X Laemmli buffer (Bio-Rad) at 95°C for 5 minutes.

Supplementary Figure 1

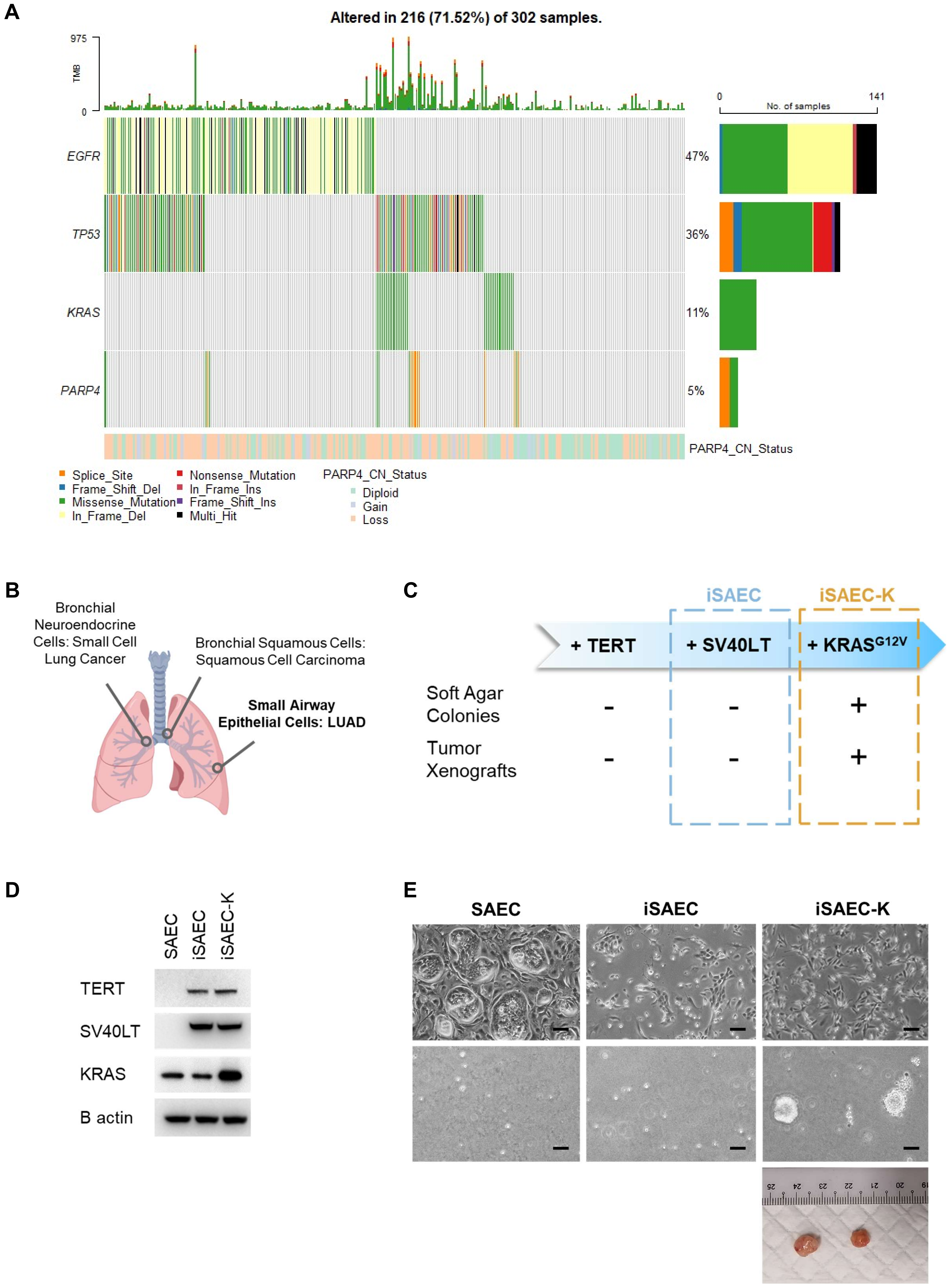

Supplementary Figure 1 cont'd

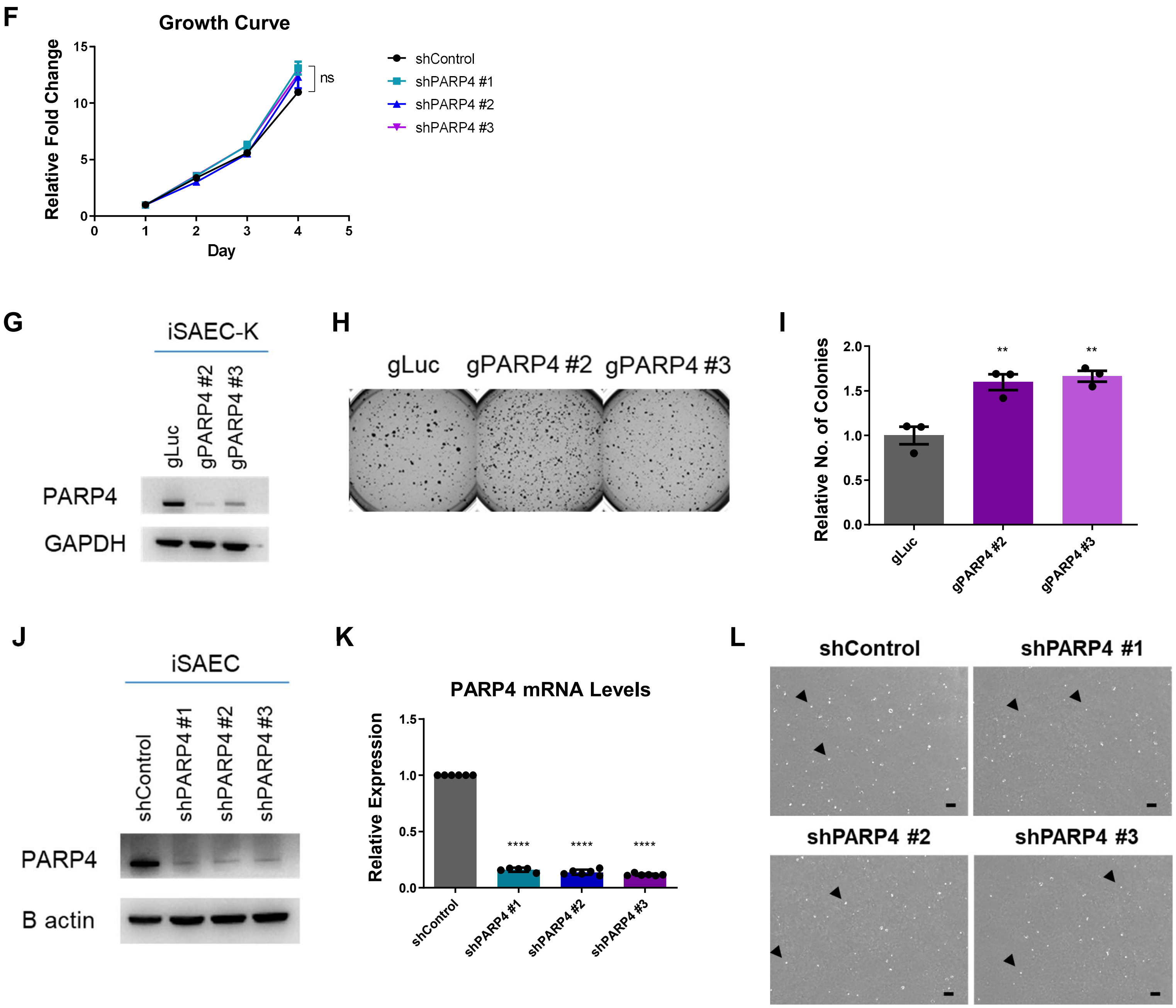

Fig. S1. Model systems to study PARP4 function

A) OncoPrint depicting the mutational profiles of EGFR, TP53, KRAS and PARP4 in the Asian LUAD cohort [4,5]. PARP4 copy number status is annotated at the bottom. **B)** Schematic diagram of the human lung and the different cellular origins of lung cancer. **C)** Schematic diagram describing the gain in tumorigenic properties of primary small airway epithelial cells (SAEC) following the cumulative addition of genetic elements. **D)** Immunoblot validation of TERT, SV40 large T antigen and KRAS overexpression in SAEC cells. **E)** Representative brightfield images of SAEC, iSAEC and iSAEC-K maintained in culture (top). Representative brightfield images of soft agar colonies formed after 2 weeks. SAEC and iSAEC remained as single cells even after two months (middle). Images taken at 4x magnification. Scale bar represents 100 μm. Representative images of tumors formed by subcutaneous injection of 1 million iSAEC-K cells after 12 weeks (bottom). **F)** Growth curves comparing the proliferative capacity of iSAEC-K control and PARP4 knockdown cells as measured by the CellTiter-Glo assay. Data represent the mean ± s.e.m., $n \geq 3$; ns: not significant, as determined by ordinary one-way ANOVA followed by the Dunnett test to correct for multiple comparisons. The multiplicity adjusted p value was reported. **G)** Immunoblot confirming the reduction in PARP4 levels using the pooled CRISPR knockout method. **H)** Representative images of soft agar colonies stained with crystal violet. **I)** Quantification of soft agar colonies. Data represent the mean ± s.e.m., n = 3; **$p<0.01$, as determined by ordinary one-way ANOVA followed by the Dunnett test to correct for multiple comparisons. The multiplicity adjusted p value was reported. **J)** Immunoblot indicating knockdown of PARP4 in a primary lung line expressing TERT and SV40 LT (iSAEC). **K)** RT-qPCR analysis indicating a significant reduction in PARP4 transcript levels in knockdown cells. Data represent the mean ± s.e.m., n = 6; ****$p<0.0001$, as determined by ordinary one-way ANOVA followed by the Dunnett test to correct for multiple comparisons. The multiplicity adjusted p value was reported. **L)** Representative brightfield images of cells in soft agar at 8 weeks, taken at 4x magnification. Scale bar represents 100 μm. Control and PARP4 knockdown cells remained as single cells (examples indicated by black arrowheads) and did not proliferate under anchorage-independent conditions. Data represent the mean ± s.e.m., n = 3; ****$p<0.0001$, as determined by ordinary one-way ANOVA followed by the Dunnett test to correct for multiple comparisons. The multiplicity adjusted p value was reported.

Supplementary Figure 2

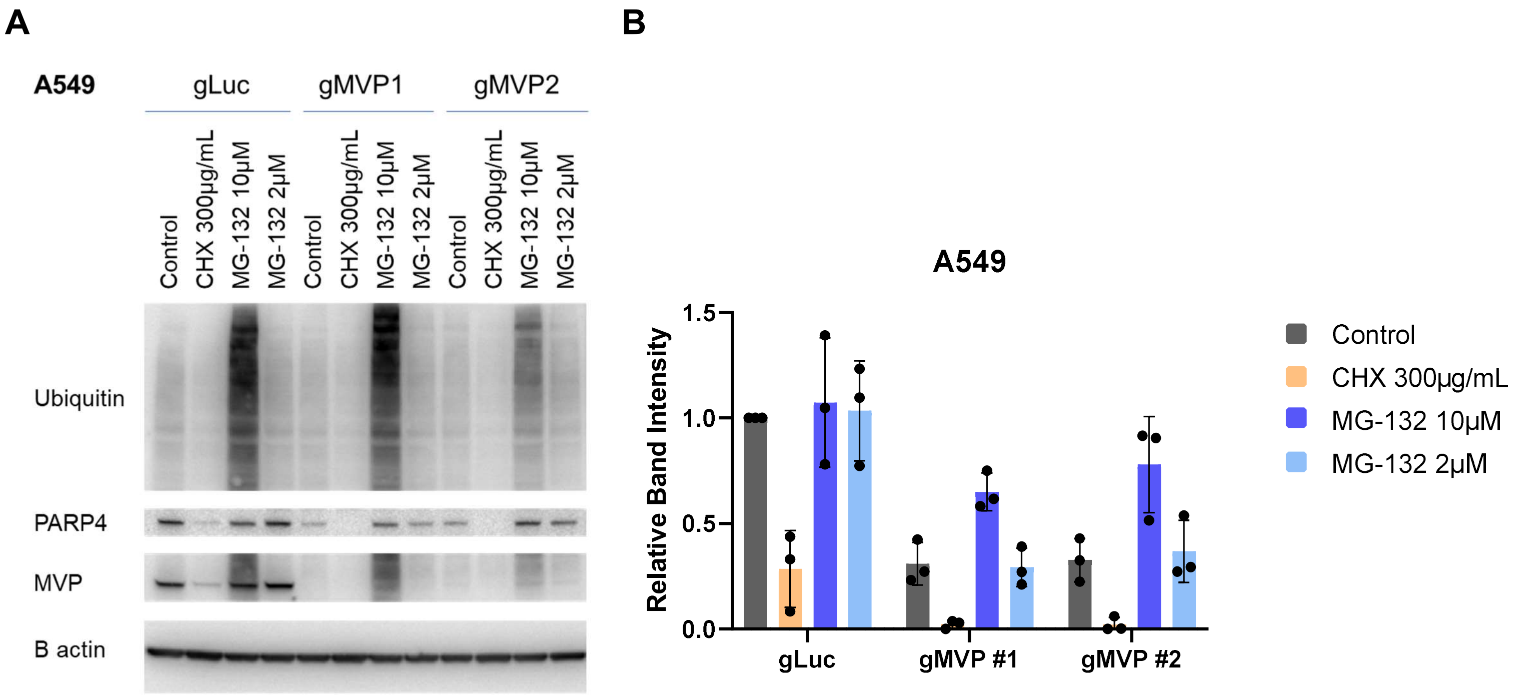

Fig. S2. Effect of MVP on PARP4 protein levels

A) Immunoblot depicting PARP4 protein levels in control and MVP-depleted A549 cells at steady state and following 24h treatment with the indicated concentrations of cycloheximide (CHX) or MG-132. **B)** Quantification of PARP4 band intensity normalized to B actin across three independent biological replicates. Data represent the mean ± s.d., n = 3.

Supplementary Figure 3

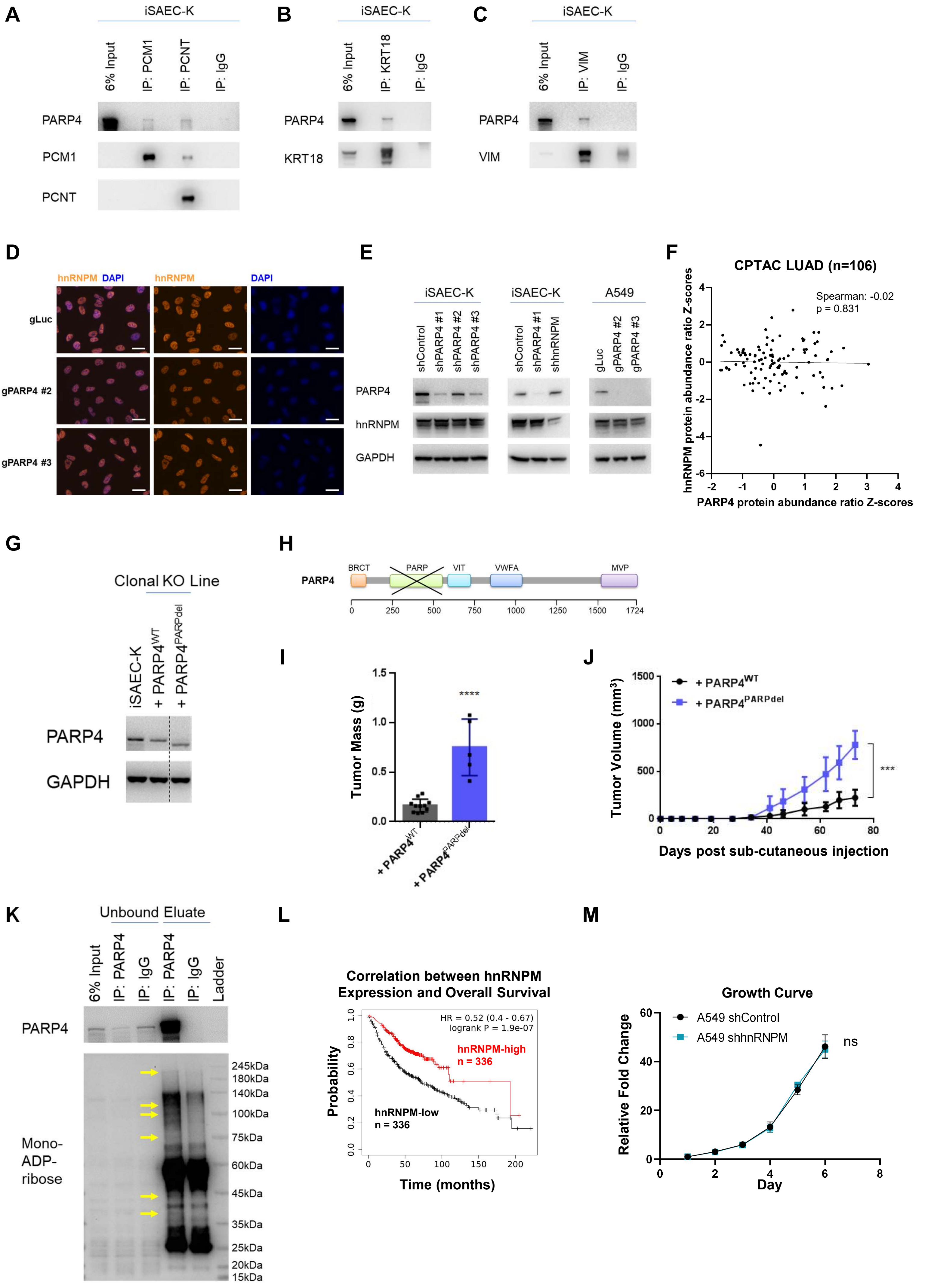

Fig. S3. PARP4 and its putative binding partners

A) Immunoblot analysis following immunoprecipitation of PCM1 and PCNT in iSAEC-K cells. **B)** Immunoblot analysis following immunoprecipitation of KRT18 in iSAEC-K cells. **C)** Immunoblot analysis following immunoprecipitation of VIM in iSAEC-K cells. **D)** Representative immunofluorescence images of A549 gLuc, gPARP4#2 and gPARP4#3 cells stained with hnRNPM antibody (orange) and DAPI (blue). Images were taken using the Zeiss Observer Z1 microscope. Scale bar, 100 µm. **E)** Immunoblot comparing PARP4 and hnRNPM expression levels upon PARP4 or hnRNPM depletion in iSAEC-K and A549 cells. **F)** Plot of protein abundance ratio Z-scores of hnRNPM against PARP4 from the CPTAC LUAD cohort (n=106) [6]. Spearman correlation coefficient and p-value indicated. Data retrieved from cBioportal [7]. **G)** Immunoblot analysis of PARP4 levels in iSAEC-K PARP4 clonal knockout cells expressing wildtype or PARP domain-deleted PARP4. **H)** Schematic diagram of PARP4 protein structure. **I)** Bar graph comparing mass of tumors formed after 8 weeks from the subcutaneous injection of 1 million iSAEC-K cells per flank of NSG mice. **J)** Growth curve of tumors formed in **I)**. Tumor volume was calculated as 0.5 x length x width^2. Data represent the mean ± s.d., n ≥ 5. **K)** Immunoblot comparing mono-ADP-ribose bands between IgG control and PARP4 eluates from immunoprecipitation performed on iSAEC-K lysates. Bands specific to PARP4 eluates are indicated with yellow arrows. **L)** Kaplan-Meier plot generated using LUAD microarray data (n=672, Affymetrix ID 1555844_s_at for hnRNPM) from the KM Plotter database, where data was aggregated from multiple cohorts across 12 GEO datasets [8,9]. The patient group with high gene expression (n=336) is displayed in red, while the low expression group (n=336) is marked in black. **M)** Proliferative capacity of A549 shControl and shhnRNPM cells as measured by the CellTiter-Glo assay. Data represent the mean ± s.e.m., n = 8; ***p<0.001, ****p<0.0001, ns: not significant, as determined by unpaired two-tailed t-test.

Supplementary Figure 4

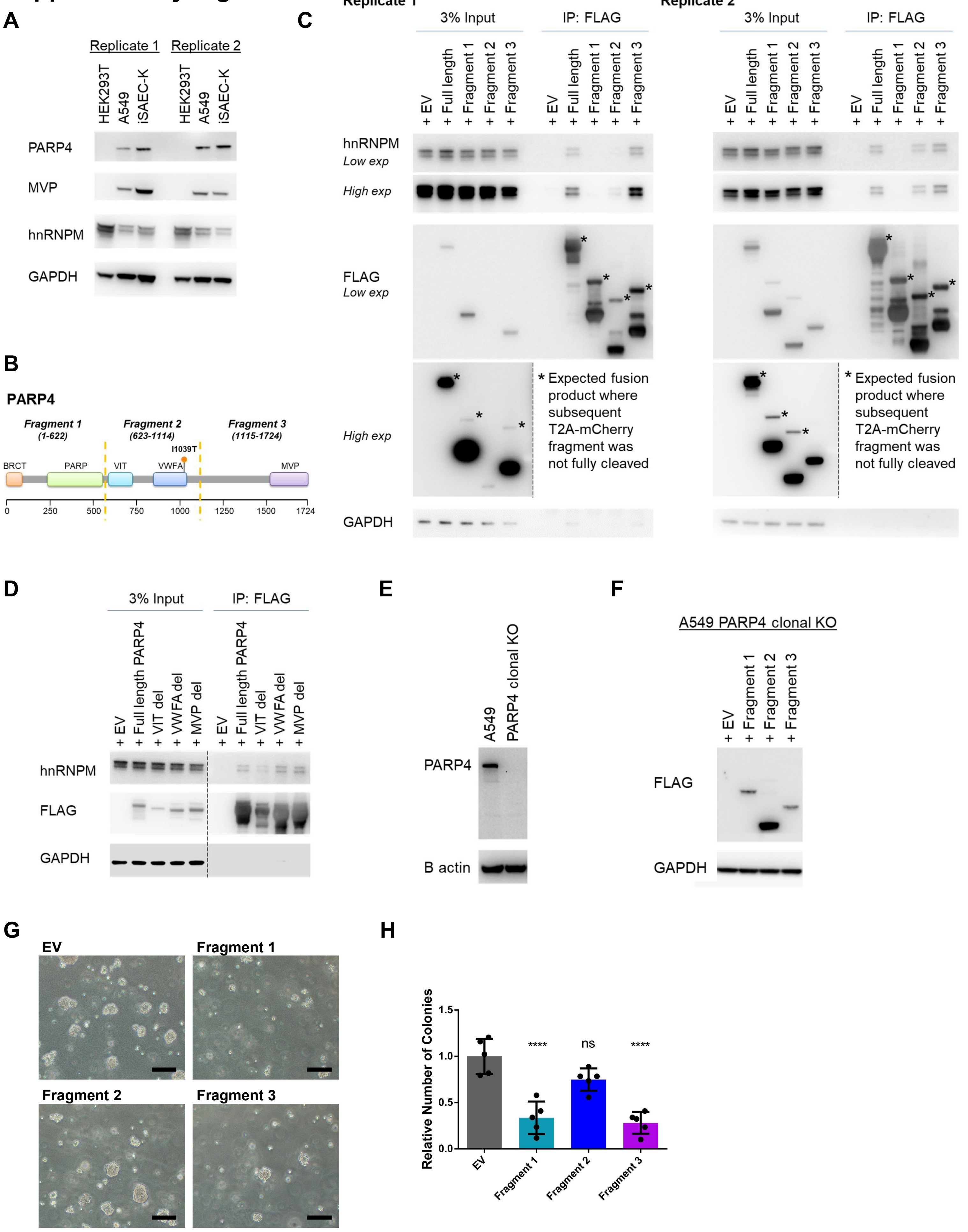

Fig. S4. Nature of PARP4-hnRNPM interaction

A) Immunoblot comparing PARP4, MVP and hnRNPM expression levels among HEK293T and the A549 and iSAEC-K lung cancer cells. **B)** Schematic diagram of PARP4 protein structure depicting the domains contained within the 3 fragments. **C)** Immunoblots depicting 2 independent replicates of FLAG pulldown on lysates of HEK293T transiently transfected with FLAG-tagged full length PARP4 or PARP4 fragments. Asterisks indicate bands corresponding to the expected fusion product where the subsequent T2A-mCherry fragment had not been fully cleaved. We note that in replicate 1 (left), expression levels of PARP4-Fragment 2 were lower than that of the other fragments. Expression across the fragments was more even in a second replicate (right). **D)** Immunoblot depicting expression of FLAG-tagged PARP4 domain deletion mutants in transiently transfected HEK293T and co-immunoprecipitation of hnRNPM upon FLAG pull down in all conditions except empty vector control. **E)** Immunoblot depicting loss of PARP4 protein in an A549 PARP4 clonal knockout (CKO) cell line. **F)** Immunoblot depicting expression levels of FLAG-tagged PARP4-Fragments 1-3 in the A549 PARP4 CKO cells. **G)** Representative brightfield images of soft agar colonies formed by the A549 PARP4 CKO cells after 3 weeks. Images taken at 4x magnification. Scale bar represents 100 μm. **H)** Quantitation of soft agar colonies formed by A549 PARP4 CKO cells after 3 weeks. Random fields of view across independent replicates were captured using a brightfield microscope at 4x magnification. Colonies at least 25 μm in diameter were counted and quantified relative to the empty vector control. Data represent the mean ± s.e.m., n = 5; ****$p<0.0001$, ns: not significant, as determined by ordinary one-way ANOVA followed by the Dunnett test to correct for multiple comparisons to a control group. The multiplicity adjusted p value was reported.

Supplementary Figure 5

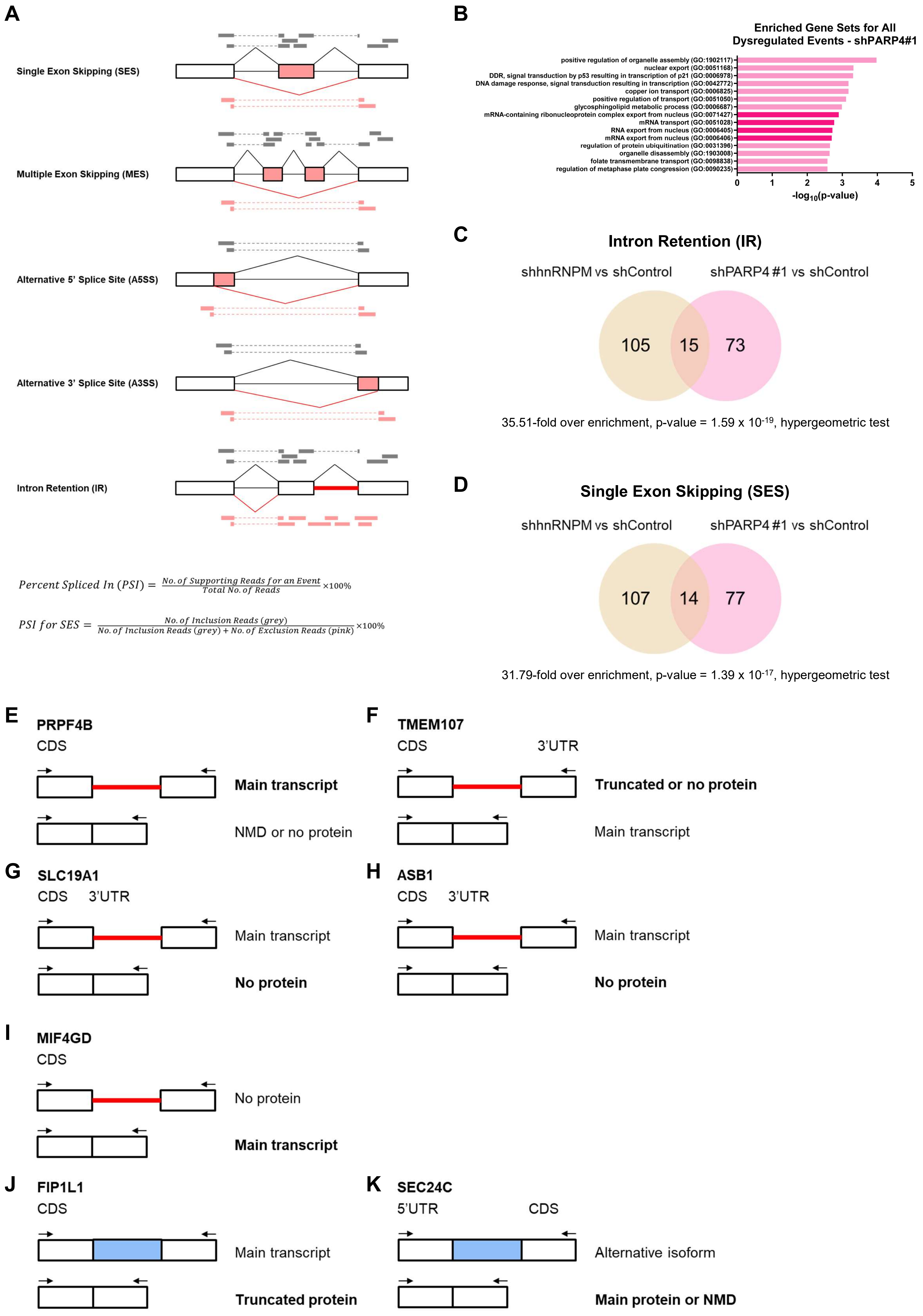

Fig. S5. Outcomes of alternative splicing

A) Schematic diagram of each of the five splice event types recognized by the PSI-Sigma pipeline (top). Exons represented by the boxes are connected by lines representing the introns. The alternatively spliced feature is highlighted in pink or red. Examples of sequencing reads that support the constitutive event are represented in grey, while reads that support the alternative event are represented in pink. A splice event must be represented by a minimum of five supporting sequencing reads before it is successfully detected by the pipeline. Calculation of the Percent Spliced In (PSI) value (bottom). For each splice event, the pipeline computes the PSI value, which represents the percentage of sequencing reads supporting a particular splice feature out of total reads at the event region. As an example, the PSI value for a variable exon (in pink) for an SES event would be determined by the percentage of sequencing reads supporting the inclusion of that exon (grey reads), out of the total number of reads for the splice region that either include (grey reads) or exclude the exon (pink reads). **B)** Top 15 enriched GO Biological Process 2021 gene sets among genes with significantly dysregulated splicing upon PARP4 knockdown ($|\Delta PSI| > 10$, p value < 0.05). Gene sets related to RNA metabolism and splicing are highlighted with a darker shade. **C, D)** Overlap in genes with significantly dysregulated ($|\Delta PSI| > 10$, p value < 0.05) **C)** IR or **D)** SES identified from the iSAEC-K shhnRNPM versus shControl analysis and the iSAEC-K shPARP4 #1 versus shControl analysis, with **C)** 35.51-fold over enrichment and p-value = 1.59×10^{-19}, or **D)** 31.79-fold over enrichment and p-value = 1.39×10^{-17}, as determined by hypergeometric test. **E-K)** Summary of outcomes of dysregulated splicing upon hnRNPM knockdown for the validated IR **(E-I)** and SES **(J, K)** events. The expected outcome upon hnRNPM loss is highlighted in bold.

Table S1. Distribution of PARP4 I1039T mutation across different cancer cohorts

Data retrieved from the International Cancer Genome Consortium (ICGC) Data Portal [10,11]

Project	Tumor Subtype	No. of PARP4 I1039T cases	Cohort size	Frequency of PARP4 I1039T (%)
Colorectal Cancer (China)	Adenocarcinoma, non-Western	9	321	2.80
Lung Cancer (Korea)	Adenocarcinoma, Squamous cell carcinoma	4	170	2.35
Skin Cancer (Australia)	Melanoma	3	183	1.64
Acute Myeloid Leukemia (Korea)	Acute myeloid leukemia	2	205	0.98
Gastric Cancer (China)	Intestinal- and diffuse-type	1	123	0.81
Liver Cancer (China)	Hepatocellular carcinoma HBV-associated	3	402	0.75
Esophageal Cancer (China)	Squamous carcinoma	1	332	0.30

Table S2. List of candidates of interest identified by mass spectrometry with normalized forward SILAC ratio > 1.1 and normalized reverse SILAC ratio < 0.9

<table>
<tr><th>Gene Name</th><th>Description</th><th>Normalized Forward H/L Ratio</th><th>Normalized Reverse H/L Ratio</th></tr>
<tr><td>MVP</td><td>Major vault protein</td><td>7.95</td><td>0.12</td></tr>
<tr><td>PARP4</td><td>Poly (ADP-ribose) polymerase 4</td><td>6.06</td><td>0.19</td></tr>
<tr><td>PHB2</td><td>Prohibitin-2</td><td>1.60</td><td>0.87</td></tr>
<tr><td>PHB</td><td>Prohibitin</td><td>1.52</td><td>0.78</td></tr>
<tr><td>KRT18</td><td>Keratin 18</td><td>1.50</td><td>0.86</td></tr>
<tr><td>PYGB</td><td>Glycogen phosphorylase, brain form</td><td>1.46</td><td>0.43</td></tr>
<tr><td>VIM</td><td>Vimentin</td><td>1.45</td><td>0.37</td></tr>
<tr><td>RPS27A</td><td>Ubiquitin-40S ribosomal protein S27a; Ubiquitin; 40S ribosomal protein S27a</td><td rowspan="4">1.33</td><td rowspan="4">0.53</td></tr>
<tr><td>UBC</td><td>Polyubiquitin-C; Ubiquitin</td></tr>
<tr><td>UBB</td><td>Polyubiquitin-B; Ubiquitin</td></tr>
<tr><td>UBA52</td><td>Ubiquitin-60S ribosomal protein L40; Ubiquitin; 60S ribosomal protein L40</td></tr>
<tr><td>PCNT</td><td>Pericentrin</td><td>1.33</td><td>0.85</td></tr>
<tr><td>DDX3X</td><td>ATP-dependent RNA helicase DDX3X</td><td rowspan="2">1.30</td><td rowspan="2">0.86</td></tr>
<tr><td>DDX3Y</td><td>ATP-dependent RNA helicase DDX3Y</td></tr>
<tr><td>PCM1</td><td>Pericentriolar material 1 protein</td><td>1.19</td><td>0.76</td></tr>
<tr><td>ACBD5</td><td>Acyl-CoA-binding domain-containing protein 5</td><td>1.17</td><td>0.84</td></tr>
<tr><td>HNRNPM</td><td>Heterogeneous nuclear ribonucleoprotein M</td><td>1.14</td><td>0.77</td></tr>
</table>

Table S3. Top 15 upregulated IR events in iSAEC-K shhnRNPM versus shControl

Gene	Target Region (GRCh38 coordinates)	ΔPSI	p-value
SIAH1	chr16:48361760-48361853	47.37	0.028
IP6K2	chr3:48694241-48694701	29.94	0.018
DPY19L4	chr8:94792358-94792730	29.60	0.041
SYVN1	chr11:65128489-65128562	29.53	0.014
IP6K2	chr3:48694240-48695088	29.51	0.011
IP6K2	chr3:48694825-48695089	28.25	0.005
AP5M1	chr14:57288972-57289875	28.05	0.020
MECP2	chrX:154031287-154031395	27.23	0.009
SRRM2	chr16:2770228-2770351	26.08	0.024
PHKG2	chr16:30756960-30760118	25.52	0.008
SHISA5	chr3:48468983-48469022	25.39	0.010
TMEM107	chr17:8174285-8174525	22.16	0.006
ERRFI1	chr1:8012957-8013241	21.48	0.042
CERS2	chr1:150965744-150965831	21.38	0.014
ERRFI1	chr1:8012956-8013357	21.34	0.044

Table S4. Top 15 downregulated IR events in iSAEC-K shhnRNPM versus shControl

Gene	Target Region (GRCh38 coordinates)	ΔPSI	p-value
SLC19A1	chr21:45513559-45514895	-33.04	0.002
POT1	chr7:124822552-124823970	-18.22	0.033
ASB1	chr2:238450260-238451784	-17.43	0.009
NGRN	chr15:90265877-90266287	-16.27	0.048
DCTN5	chr16:23672198-23675193	-14.57	0.037
TOR2A	chr9:127732279-127732563	-13.73	0.019
AP003352.1	chr8:98041860-98042653	-13.30	0.001
DYNC1H1	chr14:102032468-102033064	-13.20	0.007
FGF2	chr4:122893226-122897623	-12.44	0.017
IFT27	chr22:36775795-36776030	-12.14	0.030
MIF4GD	chr17:75267631-75268082	-11.43	0.042
ASB1	chr2:238446656-238451784	-11.33	0.029
AP003352.1	chr8:98041859-98042652	-10.94	0.013
ACO2	chr22:41527421-41527900	-10.51	0.030
NEMF	chr14:49782595-49782829	-10.45	0.035

Table S5. Top 15 upregulated SES events in iSAEC-K shhnRNPM versus shControl

Gene	Target Region (GRCh38 coordinates)	ΔPSI	p-value
KIAA1191	chr5:176359481-176359567	-37.92	0.006
FIP1L1	chr4:53414615-53414722	-23.32	0.019
ALDOC	chr17:28575965-28576020	-21.07	0.033
GNB1L	chr22:19821228-19821375	-17.81	0.042
SNHG1	chr11:62854888-62854938	-16.84	0.049
CHID1	chr11:893427-893519	-16.56	0.042
YBEY	chr21:46291334-46291462	-15.87	0.004
WASHC2C	chr10:45786612-45786674	-15.57	0.026
CDK7	chr5:69252418-69252451	-15.41	0.027
CA12	chr15:63328098-63328130	-15.05	0.050
AP2B1	chr17:35670857-35670898	-14.10	0.022
RPAIN	chr17:5425971-5426299	-13.98	0.044
C1orf52	chr1:85258935-85259061	-13.84	0.027
SEC24C	chr10:73746630-73746716	-13.56	0.002
CHCHD7	chr8:56212762-56212889	-13.29	0.044

Table S6. Top 15 downregulated SES events in iSAEC-K shhnRNPM versus shControl

Gene	Target Region (GRCh38 coordinates)	ΔPSI	p-value
SNHG17	chr20:38422092-38422241	34.27	0.025
POGZ	chr1:151440928-151441086	29.47	0.036
NAP1L4	chr11:2989129-2989271	28.14	0.026
ZMYND8	chr20:47212642-47212725	27.77	0.029
MRPL33	chr2:27774424-27774530	27.49	0.018
APLP2	chr11:130123612-130123779	26.51	0.021
PAK1	chr11:77397027-77397093	25.05	0.001
KIF23	chr15:69425282-69425323	23.61	0.003
HNRNPR	chr1:23340852-23341020	22.60	0.008
NCAPG2	chr7:158650832-158650972	21.36	0.001
RABGEF1	chr7:66768831-66769025	20.60	0.044
TMEM11	chr17:21210936-21211227	20.22	0.049
ANKRD11	chr16:89431256-89431326	16.61	0.007
INTS14	chr15:65607159-65607442	15.94	0.039
HP1BP3	chr1:20780345-20780540	15.50	0.010

Table S7. shRNA target sequences

Gene	shRNA Name	Target Sequence
Non-target control	shControl	CAACAAGATGAAGAGCACCAA
PARP4	shPARP4 #1	CCTGGGACTATTGGCTAAGAA
PARP4	shPARP4 #2	GCATTCAATCTCTAGGTGTAA
PARP4	shPARP4 #3	GCTCAGTACAAGTATCAAGTA
MVP	shMVP #1	CCCATCAACCTCTTCAACACA
MVP	shMVP #3	CACTTTCGATGACTTCCATAA
hnRNPM	shhnRNPM	CTGTGCAAGCTATATCTATGT

Table S8. gRNA target sequences

Gene	gRNA Name	Target Sequence
Luciferase	gLuc	ACAACTTTACCGACCGCGCC
PARP4	gPARP4 #2	CTGGGTTTGCAATATGAACG
PARP4	gPARP4 #3	AGGAGGTGGTGTGATGTCCA
MVP	gMVP #1	CATGGATATAGTGGTATGGG
MVP	gMVP #2	GGCATCCCGAGACACAGGGT

Table S9. Primer sequences for RT–qPCR analysis

Gene	Forward Primer	Reverse Primer
GAPDH	TGGCAAATTCCATGGCACCG	CGCCCCACTTGATTTTGGAGG
hnRNPM	GAGGCCATGCTCCTGGG	TTTAGCATCTTCCATGTGAAATCG
MVP	AACTCCCAAGCCCCACCC	GGGGAGCATCTAGAAGTGCAG
PARP4	CAGACGTCGGAAACCTTGGA	AACTCCATGCACACTGTCGT

Table S10. Details of primary and secondary antibodies

Primary Antibody	Company	Catalog Number
Anti-B actin	Santa Cruz Biotechnology	sc-47778
Anti-FLAG tag	Cell Signaling	CST14793S
Anti-GAPDH	Santa Cruz Biotechnology	sc-47724
Anti-H3	Abcam	ab1791
Anti-hnRNPM	Santa Cruz Biotechnology	sc-20002
Anti-KRAS	Santa Cruz Biotechnology	sc-30
Anti-KRT18	Santa Cruz Biotechnology	sc-6259
Anti-mono-ADP-ribose	Merck	MABE1076
Anti-MVP	Santa Cruz Biotechnology	sc-23916
Anti-PARP4	Santa Cruz Biotechnology	sc-515898
Anti-PCM1	Santa Cruz Biotechnology	sc-398365
Anti-PCNT	Santa Cruz Biotechnology	sc-376111
Anti-SV40 T Antigen	Santa Cruz Biotechnology	sc-55461
Anti-TERT	Santa Cruz Biotechnology	sc-393013
Anti-Ubiquitin	Cell Signaling	CST3933S
Anti-VIM	Santa Cruz Biotechnology	sc-32322
**Secondary Antibody**	**Company**	**Catalog Number**
Anti-mouse IgG, HRP-linked	Cell Signaling	CST7076S
Anti-rabbit IgG, HRP-linked	Cell Signaling	CST7074S

Table S11. Antibodies used for IP

IP Antibody	Company	Catalog Number
Anti-KRT18	Santa Cruz Biotechnology	sc-6259
Anti-PARP4	Santa Cruz Biotechnology	sc-515898
Anti-PCM1	Santa Cruz Biotechnology	sc-398365
Anti-PCNT	Santa Cruz Biotechnology	sc-376111
Anti-VIM	Santa Cruz Biotechnology	sc-32322
Mouse IgG1 Isotype Control	Cell Signaling	CST5415S

Table S12. Primer sequences for splicing validation

Gene	Forward Primer	Reverse Primer
ASB1	GGCTGGATGAACGTGTGAC	TCAAACTGGAAACCACGAGC
FIP1L1	TGCCCTTCCATCTACAAAAGC	CTGTTCTCATTTGCCCGTCG
MIF4GD	TGGAGAAAGTGGCCAATGTG	CTCCCGAGCCTGGTACTC
PRPF4B	ACGAATTAGCATCAACCAGGC	ACTGACTACACCAAGGGCAT
SEC24C	CGAAGTCTAACCTGGATCTGG	CTGGGTAGATGGGCTGGG
SLC19A1	GGTTCCTCTCTCCCACCCTA	CCTGAGATCCGGCAACATCA
TMEM107	GTGGCCCTGTCCTTCTTCAT	CGTCCTTAGGTTCCCGTCAT

References

1. Cox J, Mann M. MaxQuant enables high peptide identification rates, individualized p.p.b.-range mass accuracies and proteome-wide protein quantification. Nat Biotechnol. 2008;26:1367–72.

2. Perez-Riverol Y, Bai J, Bandla C, García-Seisdedos D, Hewapathirana S, Kamatchinathan S, et al. The PRIDE database resources in 2022: a hub for mass spectrometry-based proteomics evidences. Nucleic Acids Res. 2022;50:D543–52.

3. Lee YF, Phua CZJ, Yuan J, Zhang B, Lee MY, Kannan S, et al. PARP4 interacts with hnRNPM to regulate splicing during lung cancer progression. ProteomeXchange Consortium PRIDE partner repository (Identifier PXD050844). 2024. Available from: https://www.ebi.ac.uk/pride/archive/projects/PXD050844

4. Chen J, Yang H, Teo ASM, Amer LB, Sherbaf FG, Tan CQ, et al. Genomic landscape of lung adenocarcinoma in East Asians. Nat Genet. 2020;52:177–86.

5. Chen J, Yang H, Teo ASM, Amer LB, Sherbaf FG, Tan CQ, et al. Genomic landscape of lung adenocarcinoma in East Asians. European Genome-phenome Archive (EGA) (accession ID EGAD00001004422). 2020. Available from: https://ega-archive.org/datasets/EGAD00001004422

6. Gillette MA, Satpathy S, Cao S, Dhanasekaran SM, Vasaikar SV, Krug K, et al. Proteogenomic Characterization Reveals Therapeutic Vulnerabilities in Lung Adenocarcinoma. Cell. 2020;182:200-225.e35.

7. Gillette MA, Satpathy S, Cao S, Dhanasekaran SM, Vasaikar SV, Krug K, et al. Proteogenomic Characterization Reveals Therapeutic Vulnerabilities in Lung Adenocarcinoma. cBioPortal (CPTAC, Cell 2020). 2020. Available from: https://www.cbioportal.org/study/summary?id=luad_cptac_2020

8. Győrffy B. Transcriptome-level discovery of survival-associated biomarkers and therapy targets in non-small-cell lung cancer. Br J Pharmacol. 2024;181:362–74.

9. Győrffy B. Transcriptome-level discovery of survival-associated biomarkers and therapy targets in non-small-cell lung cancer. Kaplan-Meier Plotter (Lung Adenocarcinoma). 2024. Available from: https://kmplot.com/analysis/index.php?p=service&cancer=lung

10. Zhang J, Bajari R, Andric D, Gerthoffert F, Lepsa A, Nahal-Bose H, et al. The International Cancer Genome Consortium Data Portal. Nat Biotechnol. 2019;37:367–9.

11. Zhang J, Bajari R, Andric D, Gerthoffert F, Lepsa A, Nahal-Bose H, et al. The International Cancer Genome Consortium Data Portal. 2019. Available from: https://dcc.icgc.org/
